# Supplementary material for: Prioritising cardiovascular disease risk assessment to high risk individuals based on primary care records
Source: PLoS One. 2023 Sep 29;18(9):e0292240. doi: 10.1371/journal.pone.0292240 (PMC10540947; doi:10.1371/journal.pone.0292240)
Supplement: S1 File — (PDF) [file pone.0292240.s001.pdf]

Table 1. Comparison of characteristics of 1,642,498 individuals included in the derivation and validation datasets* and 511,591 individuals with missing predictor measurements§

Characteristics	Individuals with at least 1 measurement on any of the risk predictors (n = 1,642,498)	Individuals without any measurement on the risk predictors (n = 511,591)
Age at study entry, mean (SD), years	50.89 (13.17)	51.04 (12.47)
Men, n (%)	746,386 (45.44)	309,542 (60.51)
Incidence of CVD, rate (95% CI), 1,000 person-years	7.50 (7.45, 7.54)	14.92 (14.75, 15.09)

Abbreviations: CI, confidence interval; CVD, cardiovascular disease.

* Included 1,642,498 individuals from Clinical Practice Research Datalink, Hospital Episode Statistics, and the Office for National Statistics, England, United Kingdom, 2004-2019, aged 40-85 years, without prevalent CVD and statin treatment before study entry, and had least 1 measurement on any of systolic blood pressure, total cholesterol, HDL cholesterol, or smoking status between their study entry and study exit dates.

§ The 511,591 participants not included in the study were those without prevalent CVD and statin treatment before study entry, but have no detected measurements for complete risk predictors measurement on any of systolic blood pressure, total cholesterol, HDL cholesterol, or smoking status between their study entry and study exit dates

Table 2. Key characteristics of individuals in the derivation and validation cohorts

	Derivation cohort						Validation cohort					
	Men, N=509,127			Women, N=610,926			Men, N=237,259			Women, N=285,186		
**Characteristics**	**Mean (SD) or n (%)**	**No. (%) of persons with value**	**Median (IQR) of measures per person**	**Mean (SD) or n (%)**	**No. (%) of persons with value**	**Median (IQR) of measures per person**	**Mean (SD) or n (%)**	**No. (%) of persons with value**	**Median (IQR) of measures per person**	**Mean (SD) or n (%)**	**No. (%) of persons with value**	**Median (IQR) of measures per person**
**Earliest age during follow-up, years**	50.3 (12.7)	509,127 (100)	-	51.2 (13.6)	610,926 (100)	-	50.5 (12.6)	237,259 (100)	-	51.5 (13.6)	285,186 (100)	-
**History of diabetes§**	15,035 (3)	509,127 (100)	-	14,081 (2)	610,926 (100)	-	6,901 (3)	237,259 (100)	-	6,581 (2)	285,186 (100)	-
**Blood pressure-lowering medication prescriptions^**	80,896 (16)	509,127 (100)	-	138,254 (23)	610,926 (100)	-	38,334 (16)	237,259 (100)	-	65,140 (23)	285,186 (100)	-
**Current smoker, n (%)#**	132,314 (44)	299,787 (59)	3 (2-6)	121,987 (42)	289,273 (47)	4 (2-7)	60,195 (43)	138,851 (59)	3 (2-6)	54,732 (41)	134,995 (47)	4 (2-7)
**Systolic blood pressure mm Hg, mean (SD)#**	136.9 (18.5)	475,990 (93)	4 (2-11)	131.8 (20.3)	592,353 (97)	6 (3-14)	136.3 (18.4)	223,239 (94)	4 (2-11)	131.2 (20.3)	277,722 (97)	6 (3-14)
**Total cholesterol mmol/litre, mean (SD)#**	5.4 (1.0)	364,435 (72)	2 (1-5)	5.6 (1.0)	427,394 (70)	2 (1-5)	5.4 (1.0)	168,185 (71)	2 (1-5)	5.6 (1.1)	196,795 (69)	2 (1-5)
**HDL cholesterol mmol/litre, mean (SD)#**	1.3 (0.4)	337,436 (66)	2 (1-4)	1.6 (0.4)	395,568 (65)	2 (1-4)	1.3 (0.4)	152,567 (64)	2 (1-4)	1.6 (0.4)	178,274 (63)	2 (1-4)

* Included 1,642,498 individuals from Clinical Practice Research Datalink, Hospital Episode Statistics, and the Office for National Statistics, England, United Kingdom, 2004-2019, aged 40-85 years, without prevalent CVD and statin treatment before study entry, and had least 1 measurement on any of systolic blood pressure, total cholesterol, HDL cholesterol, or smoking status between their study entry and study exit dates.

§Defined as ever having recorded diagnosis of diabetes before the study entry

^Defined as ever having recorded use of blood-pressure lowering medications before the study entry

#Proportion or mean (standard deviation) of the first ever measurement

Table 3. Fixed intercepts and slopes from the age- and sex-specific multivariate mixed-effects models in derivation dataset

Men	Fixed intercepts						Fixed slopes			
Age	SBP	Total cholesterol	HDL cholesterol	Current smoker	Hypertension treatment	statin use	SBP	Total cholesterol	HDL cholesterol	Current smoker
40	-0.557	0.337	-0.178	0.549	0.067	-0.764	-0.001	0.011	0.000	-0.015
41	-0.543	0.351	-0.162	0.539	0.063	-0.808	-0.002	0.009	0.001	-0.015
42	-0.524	0.370	-0.147	0.527	0.061	-0.810	-0.002	0.007	0.002	-0.016
43	-0.506	0.390	-0.125	0.520	0.059	-0.828	-0.002	0.006	0.005	-0.016
44	-0.490	0.408	-0.115	0.512	0.046	-0.853	-0.002	0.004	0.005	-0.015
45	-0.471	0.430	-0.108	0.506	0.032	-0.883	-0.003	0.003	0.005	-0.015
46	-0.454	0.443	-0.097	0.499	0.020	-0.900	-0.004	0.001	0.004	-0.014
47	-0.437	0.461	-0.087	0.491	0.019	-0.910	-0.005	0.000	0.005	-0.014
48	-0.415	0.476	-0.074	0.482	0.012	-0.924	-0.005	-0.002	0.004	-0.014
49	-0.388	0.487	-0.060	0.473	0.001	-0.937	-0.004	-0.004	0.005	-0.014
50	-0.362	0.493	-0.045	0.465	-0.011	-0.949	-0.004	-0.005	0.004	-0.013
51	-0.336	0.501	-0.037	0.452	-0.027	-0.952	-0.004	-0.006	0.003	-0.014
52	-0.311	0.503	-0.025	0.441	-0.036	-0.964	-0.005	-0.008	0.003	-0.014
53	-0.283	0.506	-0.012	0.427	-0.044	-0.978	-0.006	-0.010	0.003	-0.014
54	-0.257	0.509	-0.003	0.414	-0.049	-0.993	-0.006	-0.011	0.002	-0.015
55	-0.224	0.501	0.011	0.401	-0.060	-0.995	-0.007	-0.014	0.002	-0.015
56	-0.196	0.494	0.021	0.391	-0.072	-0.999	-0.009	-0.017	0.002	-0.015
57	-0.169	0.495	0.035	0.378	-0.084	-1.004	-0.011	-0.017	0.002	-0.015
58	-0.148	0.493	0.046	0.362	-0.086	-1.020	-0.013	-0.019	0.002	-0.015
59	-0.122	0.490	0.059	0.351	-0.100	-1.031	-0.014	-0.020	0.002	-0.015
60	-0.095	0.482	0.074	0.336	-0.106	-1.035	-0.015	-0.022	0.003	-0.015
61	-0.069	0.476	0.091	0.322	-0.117	-1.045	-0.016	-0.022	0.003	-0.015
62	-0.052	0.472	0.097	0.307	-0.127	-1.057	-0.019	-0.022	0.002	-0.015
63	-0.034	0.467	0.104	0.293	-0.134	-1.061	-0.021	-0.022	0.001	-0.016
64	-0.023	0.456	0.109	0.278	-0.145	-1.069	-0.024	-0.023	0.001	-0.016
65	-0.010	0.447	0.118	0.265	-0.155	-1.073	-0.026	-0.023	0.000	-0.015
66	0.004	0.438	0.121	0.249	-0.159	-1.070	-0.027	-0.023	0.000	-0.016
67	0.012	0.428	0.128	0.238	-0.163	-1.075	-0.029	-0.023	0.000	-0.015
68	0.027	0.419	0.140	0.223	-0.169	-1.080	-0.029	-0.023	0.000	-0.015
69	0.035	0.406	0.143	0.208	-0.170	-1.082	-0.031	-0.023	0.000	-0.015

70	0.045	0.393	0.153	0.199	-0.174	-1.081	-0.033	-0.023	0.001	-0.015
71	0.053	0.372	0.160	0.187	-0.167	-1.074	-0.033	-0.024	0.001	-0.014
72	0.065	0.355	0.163	0.178	-0.171	-1.077	-0.034	-0.025	0.000	-0.014
73	0.070	0.339	0.165	0.168	-0.171	-1.079	-0.036	-0.026	0.000	-0.013
74	0.089	0.323	0.169	0.159	-0.178	-1.076	-0.035	-0.025	0.001	-0.012
75	0.097	0.307	0.171	0.151	-0.180	-1.076	-0.037	-0.025	0.001	-0.012
76	0.108	0.298	0.183	0.144	-0.178	-1.081	-0.037	-0.023	0.002	-0.011
77	0.114	0.288	0.186	0.140	-0.178	-1.082	-0.038	-0.022	0.002	-0.010
78	0.121	0.268	0.195	0.136	-0.175	-1.076	-0.038	-0.022	0.003	-0.010
79	0.125	0.249	0.212	0.128	-0.174	-1.068	-0.039	-0.022	0.004	-0.009
80	0.133	0.238	0.225	0.123	-0.183	-1.069	-0.042	-0.021	0.004	-0.009
81	0.130	0.216	0.234	0.117	-0.176	-1.062	-0.044	-0.022	0.004	-0.008
82	0.132	0.201	0.254	0.110	-0.171	-1.063	-0.045	-0.022	0.005	-0.008
83	0.126	0.186	0.267	0.105	-0.165	-1.062	-0.046	-0.022	0.004	-0.007
84	0.126	0.157	0.275	0.100	-0.175	-1.051	-0.048	-0.023	0.004	-0.007
85	0.112	0.125	0.288	0.097	-0.166	-1.043	-0.050	-0.026	0.004	-0.006
Women	Fixed intercepts						Fixed slopes			
Age	SBP	Total cholesterol	HDL cholesterol	Current smoker	Hypertension treatment	statin use	SBP	Total cholesterol	HDL cholesterol	Current smoker
40	-0.845	-0.324	-0.220	0.470	0.137	-0.560	0.018	0.013	0.005	-0.013
41	-0.810	-0.319	-0.192	0.470	0.131	-0.614	0.019	0.011	0.007	-0.012
42	-0.767	-0.281	-0.177	0.472	0.117	-0.641	0.021	0.015	0.007	-0.012
43	-0.723	-0.243	-0.160	0.474	0.097	-0.667	0.023	0.017	0.009	-0.011
44	-0.680	-0.209	-0.149	0.473	0.085	-0.702	0.023	0.017	0.009	-0.011
45	-0.638	-0.168	-0.134	0.472	0.078	-0.736	0.024	0.019	0.010	-0.011
46	-0.595	-0.132	-0.110	0.472	0.063	-0.758	0.024	0.019	0.011	-0.011
47	-0.553	-0.082	-0.091	0.470	0.057	-0.779	0.024	0.022	0.012	-0.011
48	-0.511	-0.028	-0.069	0.467	0.048	-0.803	0.023	0.025	0.013	-0.011
49	-0.472	0.026	-0.045	0.465	0.039	-0.819	0.022	0.028	0.014	-0.011
50	-0.435	0.087	-0.008	0.457	0.030	-0.848	0.020	0.031	0.017	-0.012
51	-0.400	0.147	0.024	0.451	0.025	-0.880	0.018	0.033	0.018	-0.012
52	-0.365	0.215	0.054	0.441	0.019	-0.905	0.015	0.036	0.019	-0.013
53	-0.333	0.281	0.082	0.429	0.008	-0.943	0.013	0.037	0.019	-0.014
54	-0.304	0.346	0.099	0.420	0.007	-0.973	0.010	0.038	0.018	-0.014

55	-0.273	0.404	0.110	0.407	-0.001	-0.998	0.007	0.039	0.017	-0.014
56	-0.243	0.447	0.125	0.397	0.001	-1.026	0.005	0.036	0.015	-0.014
57	-0.212	0.488	0.126	0.384	-0.006	-1.053	0.002	0.033	0.012	-0.015
58	-0.179	0.522	0.132	0.370	-0.005	-1.073	0.000	0.029	0.011	-0.016
59	-0.150	0.546	0.125	0.360	-0.007	-1.096	-0.001	0.024	0.007	-0.016
60	-0.123	0.560	0.124	0.346	-0.007	-1.112	-0.003	0.019	0.005	-0.016
61	-0.092	0.565	0.123	0.335	-0.012	-1.129	-0.005	0.014	0.003	-0.016
62	-0.061	0.575	0.117	0.321	-0.022	-1.136	-0.007	0.009	0.002	-0.016
63	-0.033	0.586	0.109	0.307	-0.025	-1.151	-0.008	0.006	0.000	-0.017
64	-0.007	0.593	0.110	0.295	-0.032	-1.155	-0.009	0.003	-0.001	-0.017
65	0.022	0.592	0.113	0.281	-0.042	-1.160	-0.010	0.000	-0.001	-0.017
66	0.049	0.590	0.120	0.268	-0.048	-1.171	-0.011	-0.003	0.000	-0.017
67	0.080	0.594	0.116	0.259	-0.057	-1.181	-0.012	-0.004	0.000	-0.017
68	0.113	0.595	0.121	0.247	-0.066	-1.190	-0.013	-0.006	0.001	-0.017
69	0.142	0.594	0.128	0.239	-0.071	-1.197	-0.014	-0.008	0.003	-0.016
70	0.171	0.593	0.134	0.229	-0.074	-1.204	-0.014	-0.009	0.004	-0.016
71	0.200	0.593	0.143	0.217	-0.084	-1.205	-0.016	-0.009	0.006	-0.016
72	0.225	0.590	0.145	0.212	-0.088	-1.207	-0.018	-0.011	0.005	-0.015
73	0.252	0.582	0.152	0.208	-0.091	-1.197	-0.019	-0.013	0.007	-0.014
74	0.276	0.579	0.155	0.199	-0.097	-1.197	-0.021	-0.013	0.006	-0.013
75	0.294	0.567	0.164	0.193	-0.099	-1.196	-0.023	-0.015	0.007	-0.013
76	0.317	0.560	0.171	0.185	-0.106	-1.188	-0.024	-0.015	0.008	-0.012
77	0.338	0.559	0.183	0.179	-0.114	-1.183	-0.025	-0.015	0.008	-0.011
78	0.355	0.552	0.191	0.173	-0.118	-1.180	-0.026	-0.015	0.008	-0.011
79	0.374	0.547	0.207	0.169	-0.129	-1.181	-0.028	-0.015	0.008	-0.010
80	0.386	0.536	0.214	0.166	-0.127	-1.174	-0.029	-0.016	0.008	-0.009
81	0.399	0.521	0.221	0.156	-0.131	-1.167	-0.031	-0.017	0.007	-0.009
82	0.416	0.503	0.233	0.147	-0.133	-1.158	-0.032	-0.018	0.007	-0.008
83	0.427	0.494	0.254	0.142	-0.133	-1.158	-0.032	-0.018	0.007	-0.008
84	0.439	0.481	0.260	0.137	-0.135	-1.161	-0.033	-0.019	0.007	-0.007
85	0.434	0.468	0.273	0.122	-0.129	-1.158	-0.037	-0.020	0.008	-0.008

Abbreviations: HDL cholesterol, high-density lipoprotein cholesterol; SBP, systolic blood pressure. Derivation dataset: Clinical Practice Research Datalink, Hospital Episode Statistics, and the Office for National Statistics, England, United Kingdom, 2004-2019

Table 4. Brier score in the validation dataset.

Sex	Brier score (95% CI)
Overall	0.2684 (0.2671, 0.2697)
Men	0.3085 (0.3068, 0.3102)
Women	0.2354 (0.2340, 0.2368)

Validation dataset: Clinical Practice Research Datalink, Hospital Episode Statistics, and the Office for National Statistics, England, United Kingdom, 2004-2019

Table 5. Key characteristics of individuals in the population health modelling in UK Biobank

	Primary care records				UK Biobank baseline			
	Men, N = 49,111		Women, N = 70,026		Men, N = 49,111		Women, N = 70,026	
**Characteristics**	**Mean (SD) or n (%)**	**No. (%) of persons with value**	**Mean (SD) or n (%)**	**No. (%) of persons with value**	**Mean (SD) or n (%)**	**No. (%) of persons with value**	**Mean (SD) or n (%)**	**No. (%) of persons with value**
**Age at baseline (years)**	55.9 (8.1)	49,111 (100%)	55.9 (7.9)	70,026 (100%)	55.9 (8.1)	49,111 (100%)	55.9 (7.9)	70,026 (100%)
**History of diabetes**	487 (1)	49,111 (100%)	436 (1)	70,026 (100%)	665 (1)	48,994 (100%)	484 (1)	69,904 (100%)
**Blood pressure-lowering medication prescriptions**	6,707 (14)	49,111 (100%)	10,198 (15)	70,026 (100%)	5,694 (12)	48,548 (99%)	7,920 (11)	69,557 (99%)
**Current smoker**	4,955 (10)	49,111 (100%)	5,106 (7)	70,026 (100%)	6,084 (12)	49,029 (100%)	6,116 (9)	69,939 (100%)
**Systolic blood pressure mm Hg, mean (SD)** #	131.6 (14.1)	39,994 (81%)	127.1 (15.0)	59,491 (85%)	140.8 (17.3)	49,025 (100%)	134.8 (19.1)	69,935 (100%)
**Total cholesterol mmol/litre, mean (SD)** #	5.4 (0.9)	24,844 (51%)	5.6 (0.9)	33,195 (47%)	5.8 (1.0)	48,565 (99%)	6.0 (1.1)	68,807 (98%)
**HDL cholesterol mmol/litre, mean (SD)** #	1.4 (0.4)	21,783 (44%)	1.7 (0.4)	29,154 (42%)	1.3 (0.3)	48,474 (99%)	1.6 (0.4)	68,691 (98%)
**BMI kg/m2, mean (SD)** #	27.1 (4.3)	29,829 (61%)	26.5 (5.3)	45,606 (65%)	27.5 (4.1)	48,975 (100%)	26.8 (5.0)	69,904 (100%)
**Ethnicity – white**§	46,853 (96)	49,014 (100%)	67,110 (96)	69,928 (100%)	46,853 (96)	49,014 (100%)	67,110 (96)	69,928 (100%)
**Townsend score, mean (SD)** §	-1.5 (3.0)	49,106 (100%)	-1.5 (2.9)	70,022 (100%)	-1.5 (3.0)	49,106 (100%)	-1.5 (2.9)	70,022 (100%)
**Family history of CVD**^	1,647 (3)	49,111 (100%)	2,615 (4)	70,026 (100%)	1,647 (3)	49,111 (100%)	2,615 (4)	70,026 (100%)
**Chronic kidney disease**^	60 (0.1)	49,111 (100%)	83 (0.1)	70,026 (100%)	60 (0.1)	49,111 (100%)	83 (0.1)	70,026 (100%)
**Rheumatoid arthritis**	175 (100.0)	49,111 (100%)	369 (100.0)	70,026 (100%)	414 (0.8)	49,111 (100%)	1,080 (2)	70,026 (100%)
**Atrial fibrillation**	635 (100.0)	49,111 (100%)	1,871 (100.0)	70,026 (100%)	137 (0.3)	49,111 (100%)	94 (0.1)	70,026 (100%)

§Risk factor was only available at baseline and was used when conducting population health modelling with primary care records.

^Risk factor was only available in primary care records and was used when conducting population health modelling with baseline data.

#Mean (standard deviation) of last observed measurement before UK Biobank baseline.

Table 6. Number needed to invite to prevent one event and number of events captured when prioritising with eHEART in a hypothetical population of 100,000 individuals in England assuming 55% of those invited for formal assessment attend.

Hypothetical population of 100,000				Full formal assessment only (QRISK2 threshold=10%)		Prioritisation tool (eHEART threshold=10%) followed by full formal assessment (QRISK2 threshold=10%)			Prioritisation tool (eHEART threshold corresponding to age- and sex-specific 5% false negative rates) followed by full formal assessment (QRISK2 threshold=10%)			
Sex	Age group	Expected N	Expected CVD events in 10 years	Events captured N (%)	Number needed to invite to prevent 1 CVD event	Individuals prioritised N	Events captured N (%)	Number needed to invite to prevent 1 CVD event	Individuals prioritised N	Prioritisation threshold for eHEART	Events captured N (%)	Number needed to invite to prevent 1 CVD event
Men	40-49	17673	516	96 (18.6%)	3665.6 (2670.4, 4337.6)	598	32 (6.3%)	333.1 (139.1, 430.0)	11054	3.0%	91 (17.7%)	2190.4 (1543.8, 2609.3)
	50-59	18061	1294	844 (65.2%)	428.2 (408.6, 446.4)	6348	518 (40.0%)	223.1 (204.7, 238.2)	14576	6.2%	810 (62.6%)	327.3 (310.4, 342.0)
	60-69	14266	1756	1714 (97.6%)	166.4 (165.2, 167.6)	13391	1659 (94.5%)	146.9 (145.1, 148.4)	13391	10%	1659 (94.5%)	146.9 (145.1, 148.4)
	**Total**	**50000**	**3566**	**2654 (74.4%)**	**376.8 (370.4, 383.0)**	**20338**	**2209 (61.9%)**	**167.5 (164.4, 170.7)**	**39021**	**NA**	**2560 (71.8%)**	**277.1 (272.0, 282.0)**
Women	40-49	17488	277	17 (6.1%)	21211.2 (1278.2, 28510.2)	78	7 (2.5%)	224.9 (0.0, 332.9)	5495	2.0%	16 (5.7%)	6564.0 (0.0, 8970.7)
	50-59	17986	607	116 (19.1%)	3104.4 (2508.4, 3574.6)	427	28 (4.5%)	284.4 (137.1, 360.4)	10519	3.3%	113 (18.7%)	1691.8 (1348.2, 1954.5)
	60-69	14526	936	699 (74.7%)	415.8 (402.2, 428.8)	5174	437 (46.7%)	215.3 (200.9, 226.5)	11850	5.9%	677 (72.4%)	318.2 (306.7, 328.2)
	**Total**	**50000**	**1819**	**831 (45.7%)**	**1203.2 (1157.6, 1245.4)**	**5680**	**471 (25.9%)**	**219.3 (205.6 231.3)**	**27864**	**NA**	**805 (44.3%)**	**629.1 (603.8, 651.6)**

Age structure of hypothetical population extrapolated from Office for National Statistics, England, United Kingdom 2017. Expected events at 10 years based on extrapolation of incidence rates from CPRD, 2014-2019. Age group and sex specific prioritisation thresholds were defined as the minimum of 10% and the level such that the expected false negative rate is controlled to be 5%. All individuals have at least one CVD risk factor (systolic blood pressure, smoking, total and/or HDL cholesterol) recorded for eHEART. Statin compliance assumed to equal 50%.

Number needed to invite for full formal assessment only assumes that 50% of all individuals are formally assessed. Number needed to invite after prioritisation with eHEART assumes 55% of prioritised individuals are formally assessed. Under each prioritisation scenario, some individuals are not formally assessed and may go on to have events.

Table 7: **Number needed to screen to prevent one event, and number of events captured when prioritising with QRISK2 in a hypothetical population of 100,000 individuals in England.**

Hypothetical population of 100,000				Full formal assessment only (QRISK2 threshold=10%)		Prioritisation tool (QRISK2 prioritisation threshold=10%) followed by full formal assessment (QRISK2 threshold=10%)			Prioritisation tool (QRISK2 prioritisation threshold corresponding to age- and sex-specific 5% false negative rates) followed by full formal assessment (QRISK2 threshold=10%)			
Sex	Age group	Expected N	Expected CVD events in 10 years	Events captured N (%)	Number needed to screen to prevent 1 CVD event	Individuals prioritised N	Events captured N (%)	Number needed to screen to prevent 1 CVD event	Individuals prioritised N	Prioritisation threshold for QRISK2	Events captured N (%)	Number needed to screen to prevent 1 CVD event
Men	40-49	17673	516	96 (18.6%)	1832.8 (1335.2, 2168.8)	608	31 (6.0%)	195.5 (75.8, 255.3)	10253	4.0%	93 (18.0%)	1098.8 (785.1, 1298.7)
	50-59	18061	1294	844 (65.2%)	214.1 (204.3, 223.2)	8372	647 (50.0%)	129.4 (121.4, 136.7)	14269	7.3%	800 (61.8%)	178.5 (169.3, 186.4)
	60-69	14266	1756	1714 (97.6%)	83.2 (82.6, 83.8)	14196	1713 (97.6%)	82.9 (82.2, 83.5)	14196	10.0%	1713 (97.6%)	82.9 (82.2, 83.5)
	**Total**	**50000**	**3566**	**2654 (74.4%)**	**188.4 (185.2, 191.5)**	**23176**	**2391 (67.1%)**	**96.9 (95.2, 98.7)**	**38719**	**NA**	**2606 (73.1%)**	**148.6 (145.8, 151.2)**
Women	40-49	17488	277	17 (6.1%)	10605.6 (643.6, 14255.1)	169	4 (1.5%)	444.6 (0.0, 690.1)	5628	2.2%	17 (6.1%)	3412.8 (183.0, 4597.9)
	50-59	17986	607	116 (19.1%)	1552.2 (1254.2, 1787.3)	1087	37 (6.1%)	295.8 (179.5, 363.8)	8789	4.9%	112 (18.5%)	784.0 (631.2, 902.6)
	60-69	14526	936	699 (74.7%)	207.9 (201.1, 214.4)	8510	609 (65.0%)	139.9 (133.4, 145.0)	11316	8.2%	675 (72.1%)	167.7 (161.6, 172.8)
	**Total**	**50000**	**1819**	**831 (45.7%)**	**601.6 (578.8, 622.7)**	**9766**	**649 (35.7%)**	**150.5 (144.0, 156.5)**	**25732**	**NA**	**803 (44.2%)**	**320.2 (306.8, 332.3)**

Age structure of hypothetical population extrapolated from Office for National Statistics, England, United Kingdom 2017. Expected events at 10 years based on extrapolation of incidence rates from CPRD, 2014-2019. Age group and sex specific prioritisation thresholds were defined as the minimum of 10% and the level such that the expected false negative rate is controlled to be 5%. All individuals eligible for prioritisation with QRISK2 were also eligible for prioritisation with eHEART. Statin compliance assumed to equal 50%.

Number needed to screen based on assuming that all individuals are formally assessed. Under each scenario, some individuals are not formally assessed and may go on to have events.

Table 8. Number needed to invite to prevent one event and number of events captured when prioritising with QRISK2 in a hypothetical population of 100,000 individuals in England assuming 55% of those invited for formal assessment attend.

Hypothetical population of 100,000				Full formal assessment only (QRISK2 threshold=10%)		Prioritisation tool (QRISK2 prioritisation threshold=10%) followed by full formal assessment (QRISK2 threshold=10%)			Prioritisation tool (QRISK2 prioritisation threshold corresponding to age- and sex-specific 5% false negative rates) followed by full formal assessment (QRISK2 threshold=10%)			
Sex	Age group	Expected N	Expected CVD events in 10 years	Events captured N (%)	Number needed to invite to prevent 1 CVD event	Individuals prioritised N	Events captured N (%)	Number needed to invite to prevent 1 CVD event	Individuals prioritised N	Prioritisation threshold for QRISK2	Events captured N (%)	Number needed to invite to prevent 1 CVD event
Men	40-49	17673	516	96 (18.6%)	3665.6 (2670.4, 4337.6)	608	31 (6.0%)	355.5 (137.8, 464.2)	10253	4.0%	93 (18.0%)	1997.8 (1427.5, 12361.3)
	50-59	18061	1294	844 (65.2%)	428.2 (408.6, 446.4)	8372	647 (50.0%)	235.3 (220.7, 248.5)	14269	7.3%	800 (61.8%)	324.5 (307.8, 338.9)
	60-69	14266	1756	1714 (97.6%)	166.4 (165.2, 167.6)	14196	1713 (97.6%)	150.7 (149.5, 151.8)	14196	10.0%	1713 (97.6%)	150.7 (149.5, 151.8)
	**Total**	**50000**	**3566**	**2654 (74.4%)**	**376.8 (370.4, 383.0)**	**23176**	**2391 (67.1%)**	**176.2 (173.1, 179.5)**	**38719**	**NA**	**2606 (73.1%)**	**270.2 (265.1, 274.9)**
Women	40-49	17488	277	17 (6.1%)	21211.2 (1278.2, 28510.2)	169	4 (1.5%)	808.4 (0.0, 1254.7)	5628	2.2%	17 (6.1%)	6205.1 (332.7, 8359.8)
	50-59	17986	607	116 (19.1%)	3104.4 (2508.4, 3574.6)	1087	37 (6.1%)	537.8 (326.4, 661.5)	8789	4.9%	112 (18.5%)	1425.5 (1147.6, 1641.1)
	60-69	14526	936	699 (74.7%)	415.8 (402.2, 428.8)	8510	609 (65.0%)	254.4 (242.5, 263.6)	11316	8.2%	675 (72.1%)	304.9 (293.8, 314.2)
	**Total**	**50000**	**1819**	**831 (45.7%)**	**1203.2 (1157.6, 1245.4)**	**9766**	**649 (35.7%)**	**273.6 (261.8, 284.5)**	**25732**	**NA**	**803 (44.2%)**	**582.2 (557.8, 604.2)**

Age structure of hypothetical population extrapolated from Office for National Statistics, England, United Kingdom 2017. Expected events at 10 years based on extrapolation of incidence rates from CPRD, 2014-2019. Age group and sex specific prioritisation thresholds were defined as the minimum of 10% and the level such that the expected false negative rate is controlled to be 5%. All individuals have at least one CVD risk factor (systolic blood pressure, smoking, total and/or HDL cholesterol) recorded for eHEART. Statin compliance assumed to equal 50%.

Number needed to invite for full formal assessment only assumes that 50% of all individuals are formally assessed. Number needed to invite after prioritising with QRISK2 assumes 55% of prioritised individuals are formally assessed. Under each prioritisation scenario, some individuals are not formally assessed and may go on to have events.

Table 9. Summary of number of individuals with no primary care records available in population health modelling dataset by age group and for men and women.

Sex	Age group	Individuals N	Individuals without at least one CVD risk factor in primary care records N (%)
Men	40-49	15959	3135 (19.6%)
	50-59	19422	2631 (13.5%)
	60-69	20035	1695 (8.46%)
	**Total**	**55416**	**7461 (13.5%)**
Women	40-44	19943	2692 (13.5%)
	45-49	27190	2644 (9.80%)
	65-69	27225	2058 (7.56%)
	**Total**	**74358**	**7414 (9.97%)**

Prioritisation with eHEART requires at least one CVD risk factor of: systolic blood pressure, smoking, total and/or HDL cholesterol. Population health modelling dataset: 129,774 individuals in UK Biobank

Table 10. Number needed to screen to prevent one event and number of events captured when prioritising with eHEART, including formally assessing individuals without a primary care record, in a hypothetical population of 100,000 individuals in England.

Hypothetical population of 100,000				Full formal assessment only (QRISK2 threshold=10%)		Prioritisation tool (eHEART threshold=10%) followed by full formal assessment (QRISK2 threshold=10%)			Prioritisation tool (eHEART threshold corresponding to age- and sex-specific 5% false negative rates) followed by full formal assessment (QRISK2 threshold=10%)			
Sex	Age group	Expected N	Expected CVD events in 10 years	Events captured N (%)	Number needed to screen to prevent 1 CVD event	Individuals prioritised N	Events captured N (%)	Number needed to screen to prevent 1 CVD event	Individuals prioritised N	Prioritisation threshold for eHEART	Events captured N (%)	Number needed to screen to prevent 1 CVD event
Men	40-49	17673	447	82 (18.4%)	2139.0 (1611.8, 2528.2)	1565	30 (6.7%)	513.0 (226.0, 657.1)	11429	3.0%	78 (17.5%)	1450.2 (1067.9, 1723.9)
	50-59	18061	1214	789 (64.9%)	228.8 (219.0, 238.5)	6888	497 (40.9%)	138.4 (128.2, 147.4)	14737	6.2%	759 (62.5%)	194.1 (185.0, 202.6)
	60-69	14266	1684	1646 (97.6%)	86.7 (86.09, 87.3)	13419	1594 (94.5%)	84.2 (83.3, 85.1)	13419	10.0%	1594 (94.5%)	84.2 (83.3, 85.1)
	**Total**	**50000**	**3345**	**2518 (75.2%)**	**198.6 (195.5, 201.8)**	**21872**	**2122 (63.4%)**	**103.1 (101.1, 105.1)**	**39584**	**NA**	**2432 (72.6%)**	**162.8 (159.9, 165.8)**
Women	40-49	17488	257	15 (6.0%)	11524.4 (1349.6, 15596.6)	1159	6 (2.3%)	1986.5 (0.0, 2949.0)	6240	2.0%	14 (5.5%)	4454.6 (0.0, 6067.7)
	50-59	17986	590	111 (18.8%)	1610.7 (1326.7, 1829.0)	1246	27 (4.6%)	450.0 (231.0, 565.9)	10867	3.3%	108 (18.4%)	997.1 (815.0, 1133.2)
	60-69	14526	918	684 (74.5%)	212.5 (205.4, 218.8)	5493	432 (47.1%)	127.0 (119.7, 133.9)	11942	5.9%	663 (72.2%)	180.1 (173.4, 186.0)
	**Total**	**50000**	**1765**	**810 (45.9%)**	**616.8 (594.8, 638.3)**	**7898**	**466 (26.4%)**	**169.5 (158.6, 179.0)**	**29049**	**NA**	**785 (44.5%)**	**369.6 (355.7, 382.7)**

Age structure of hypothetical population extrapolated from Office for National Statistics, England, United Kingdom 2017. Expected events at 10 years based on extrapolation of incidence rates from CPRD, 2014-2019. Age group and sex specific prioritisation thresholds were defined as the minimum of 10% and the level such that the expected false negative rate is controlled to be 5%. Individuals with at least one CVD risk factor (systolic blood pressure, smoking, total and/or HDL cholesterol) recorded in primary care electronic health records were assessed using eHEART. Individuals without at least one risk factor were invited for full formal risk assessment. Number needed to screen based on assuming that all individuals are formally assessed. Under each scenario, some individuals are not formally assessed and may go on to have events. Statin compliance assumed to equal 50%.

Table 11. Number needed to screen to prevent one event and number of events captured when prioritising with QRISK2 in all eligible individuals, in a hypothetical population of 100,000 individuals in England.

**Hypothetical population of 100,000**				**Full formal assessment only (QRISK2 threshold=10%)**		**Prioritisation tool (QRISK2 prioritisation threshold=10%) followed by full formal assessment (QRISK2 threshold=10%)**			**Prioritisation tool (QRISK2 prioritisation threshold corresponding to age- and sex-specific 5% false negative rates) followed by full formal assessment (QRISK2 threshold=10%)**			
Sex	Age group	Expected N	Expected CVD events in 10 years	Events captured N (%)	Number needed to screen to prevent 1 CVD event	Individuals prioritised N	Events captured N (%)	Number needed to screen to prevent 1 CVD event	Individuals prioritised N	Prioritisation threshold for QRISK2	Events captured N (%)	Number needed to screen to prevent 1 CVD event
Men	40-49	17673	447	82 (18.4%)	2139.0 (1611.8, 2528.2)	1574	29 (6.4%)	538.5 (199.5, 692.5)	10673	4.0%	80 (17.8%)	1332.8 (981.8, 1584.1)
	50-59	18061	1214	789 (64.9%)	228.8 (219.0, 238.5)	8818	613 (50.5%)	143.8 (135.3, 151.4)	14443	7.4%	750 (61.7%)	192.6 (183.5, 201.1)
	60-69	14266	1684	1646 (97.6%)	86.7 (86.09, 87.3)	14198	1645 (97.6%)	86.3 (85.7, 86.9)	14198	10.0%	1645 (97.6%)	86.3 (85.7, 86.9)
	**Total**	**50000**	**3345**	**2518 (75.2%)**	**198.6 (195.5, 201.8)**	**24591**	**2288 (68.3%)**	**107.5 (105.7, 109.3)**	**39316**	**NA**	**2475 (73.9%)**	**158.9 (156.2, 161.6)**
Women	40-49	17488	257	15 (6.0%)	11524.4 (1349.6, 15596.6)	1245	4 (1.4%)	3553.8 (0.0, 5562.4)	6364	2.2%	15 (6.0%)	4193.8 (548.0, 5674.7)
	50-59	17986	590	111 (18.8%)	1610.7 (1326.7, 1829.0)	1875	36 (6.1%)	512.0 (310.0, 636.4)	9128	4.9%	108 (18.2%)	852.8 (697.0, 967.4)
	60-69	14526	918	684 (74.5%)	212.5 (205.4, 218.8)	8715	597 (65.0%)	146.0 (139.7, 151.1)	11425	8.2%	661 (72.0%)	172.9 (166.7, 178.0)
	**Total**	**50000**	**1765**	**810 (45.9%)**	**616.8 (594.8, 638.3)**	**11835**	**637 (36.1%)**	**185.7 (177.2, 192.6)**	**27007**	**NA**	**784 (44.4%)**	**344.4 (331.5, 356.8)**

Age structure of hypothetical population extrapolated from Office for National Statistics, England, United Kingdom 2017. Expected events at 10 years based on extrapolation of incidence rates from CPRD, 2014-2019. Age group and sex specific prioritisation thresholds were defined as the minimum of 10% and the level such that the expected false negative rate is controlled to be 5%. All individuals were eligible for prioritisation with QRISK2. Number needed to screen based on assuming that all individuals are formally assessed. Under each scenario, some individuals are not formally assessed and may go on to have events. Statin compliance assumed to equal 50%.

Table 12. Number needed to screen to prevent one event and number of events captured when prioritising with eHEART in all eligible individuals, in a hypothetical population of 100,000 individuals in England, assuming a 5% formal risk assessment threshold.

Hypothetical population of 100,000				Full formal assessment only (QRISK2 threshold=5%)		Prioritisation tool (eHEART threshold=5%) followed by full formal assessment (QRISK2 threshold=5%)			Prioritisation tool (eHEART threshold corresponding to age- and sex-specific 2.5% false negative rates) followed by full formal assessment (QRISK2 threshold=5%)			
Sex	Age group	Expected N	Expected CVD events in 10 years	Events captured N (%)	Number needed to screen to prevent 1 CVD event	Individuals prioritised N	Events captured N (%)	Number needed to screen to prevent 1 CVD event	Individuals prioritised N	Prioritisation threshold for eHEART	Events captured N (%)	Number needed to screen to prevent 1 CVD event
Men	40-49	17673	516	320 (62.0%)	551.6 (502.1, 594.3)	4735	189 (36.7%)	249.6 (209.4, 281.9)	15506	2.1%	318 (61.7%)	486.4 (444.6, 524.2)
	50-59	18061	1294	1254 (96.9%)	144.1 (142.4, 145.7)	16506	1211 (93.6%)	136.3 (133.9, 138.5)	17296	4.3%	1237 (95.6%)	139.8 (137.9, 141.6)
	60-69	14266	1756	1756 (100.0%)	81.3 (81.3, 81.3)	14259	1755 (99.9%)	81.3 (81.2, 81.3)	14259	5.0%	1755 (99.9%)	81.3 (81.2, 81.3)
	**Total**	**50000**	**3566**	**3330 (93.4%)**	**150.2 (148.9, 151.5)**	**35500**	**3156 (88.5%)**	**112.5 (111.4, 113.7)**	**47062**	**NA**	**3311 (92.8%)**	**142.2 (140.8, 143.5)**
Women	40-49	17488	277	61 (22.2%)	2872.3 (1950.0, 3465.7)	711	26 (9.3%)	280.4 (102.3, 364.7)	10956	1.3%	59 (21.2%)	1877.8 (1245.2, 2293.0)
	50-59	17986	607	432 (71.1%)	416.9 (395.9, 436.4)	4595	226 (37.3%)	203.3 (180.9, 221.8)	15403	2.2%	422 (69.6%)	365.0 (345.2, 382.7)
	60-69	14526	936	930 (99.4%)	156.2 (155.5, 156.8)	13410	901 (96.3%)	148.9 (147.3, 150.3)	14074	4.4%	918 (98.1%)	153.4 (152.2, 154.4)
	**Total**	**50000**	**1819**	**1423 (78.2%)**	**351.5 (344.7, 357.9)**	**18717**	**1152 (63.3%)**	**162.4 (158.6, 166.1)**	**40433**	**NA**	**1398 (76.9%)**	**289.2 (283.5, 294.8)**

Age structure of hypothetical population extrapolated from Office for National Statistics, England, United Kingdom 2017. Expected events at 10 years based on extrapolation of incidence rates from CPRD, 2014-2019. Age group and sex specific prioritisation thresholds were defined as the minimum of 5% and the level such that the expected false negative rate is controlled to be 2.5%. All individuals have at least one CVD risk factor (systolic blood pressure, smoking, total and/or HDL cholesterol) recorded for eHEART.

Number needed to screen based on assuming that all individuals are formally assessed. Under each scenario, some individuals are not formally assessed and may go on to have events. Statin compliance assumed to be equal to 50%.

Table 13: **Number needed to screen to prevent one event, and number of events captured when prioritising with QRISK2 in a hypothetical population of 100,000 individuals in England, assuming a 5% formal risk assessment threshold.**

**Hypothetical population of 100,000**				**Full formal assessment only (QRISK2 threshold=5%)**		**Prioritisation tool (QRISK2 prioritisation threshold=5%) followed by full formal assessment (QRISK2 threshold=5%)**			**Prioritisation tool (QRISK2 prioritisation threshold corresponding to age- and sex-specific 2.5% false negative rates) followed by full formal assessment (QRISK2 threshold=5%)**			
Sex	Age group	Expected N	Expected CVD events in 10 years	Events captured N (%)	Number needed to screen to prevent 1 CVD event	Individuals prioritised N	Events captured N (%)	Number needed to screen to prevent 1 CVD event	Individuals prioritised N	Prioritisation threshold for QRISK2	Events captured N (%)	Number needed to screen to prevent 1 CVD event
Men	40-49	17673	516	320 (62.0%)	551.6 (502.1, 594.3)	6302	247 (47.9%)	254.9 (220.8, 282.0)	14500	3.0%	318 (61.7%)	454.8 (413.7, 490.8)
	50-59	18061	1294	1254 (96.9%)	144.1 (142.4, 145.7)	17877	1251 (96.7%)	142.9 (141.1, 144.5)	17877	5.0%	1251 (96.7%)	142.9 (141.1, 144.5)
	60-69	14266	1756	1756 (100.0%)	81.3 (81.3, 81.3)	14266	1756 (100.0%)	81.3 (81.3, 81.3)	14266	5.0%	1756 (100.0%)	81.3 (81.3, 81.3)
	**Total**	**50000**	**3566**	**3330 (93.4%)**	**150.2 (148.9, 151.5)**	**38444**	**3254 (91.3%)**	**118.1 (116.9, 119.3)**	**46643**	**NA**	**3326 (93.3%)**	**140.3 (139.0, 141.6)**
Women	40-49	17488	277	61 (22.2%)	2872.3 (1950.0, 3465.7)	957	28 (10.2%)	342.9 (157.8, 441.5)	9253	1.7%	59 (21.2%)	1585.77 (1043.7, 1937.6)
	50-59	17986	607	432 (71.1%)	416.9 (395.9, 436.4)	8307	348 (57.3%)	239.0 (221.9, 253.7)	14523	3.2%	428 (70.5%)	339.6 (321.3, 355.3)
	60-69	14526	936	930 (99.4%)	156.2 (155.5, 156.8)	14496	930 (99.4%)	155.8 (155.2, 156.4)	14496	5.0%	930 (99.4%)	155.8 (155.2, 156.4)
	**Total**	**50000**	**1819**	**1423 (78.2%)**	**351.5 (344.7, 357.9)**	**23761**	**1306 (71.8%)**	**182.0 (178.5, 185.4)**	**38272**	**NA**	**1416 (77.9%)**	**270.2 (264.9, 275.1)**

Age structure of hypothetical population extrapolated from Office for National Statistics, England, United Kingdom 2017. Expected events at 10 years based on extrapolation of incidence rates from CPRD, 2014-2019. Age group and sex specific prioritisation thresholds were defined as the minimum of 10% and the level such that the expected false negative rate is controlled to be 5%. All individuals eligible for prioritisation with QRISK2 were also eligible for prioritisation with eHEART. Statin compliance assumed to equal 50%.

Number needed to screen based on assuming that all individuals are formally assessed. Under each scenario, some individuals are not formally assessed and may go on to have events.

Fig 1. Schematic showing the landmark age approach of model derivation.

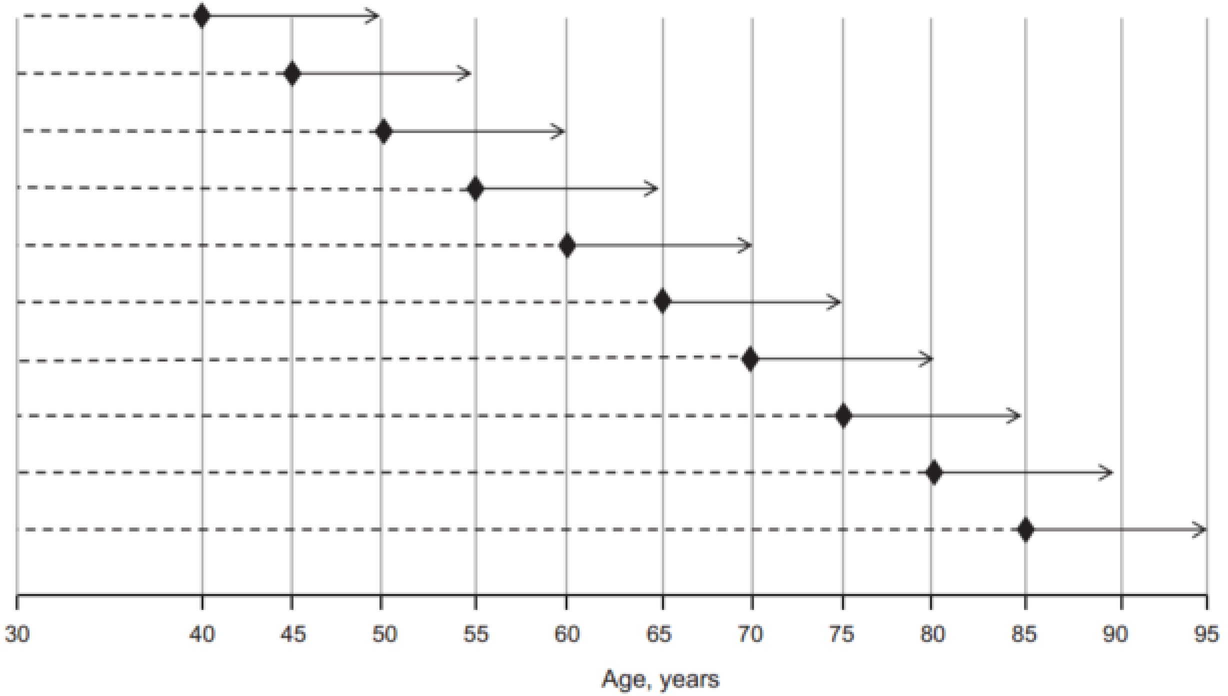

The dashed lines indicate historical repeat measures of smoking status, systolic blood pressure, total cholesterol, and high-density lipoprotein cholesterol, modeled by means of landmark-age–specific multivariate linear mixed-effects models. The diamonds show the landmark age (time of risk prediction). The arrows indicate the 10-year follow-up to the point of a cardiovascular disease event or censoring, modeled via a landmark Cox model. This figure was published in our previous work[1].

Fig 2. Flowchart showing selection of patient records for analysis in CPRD

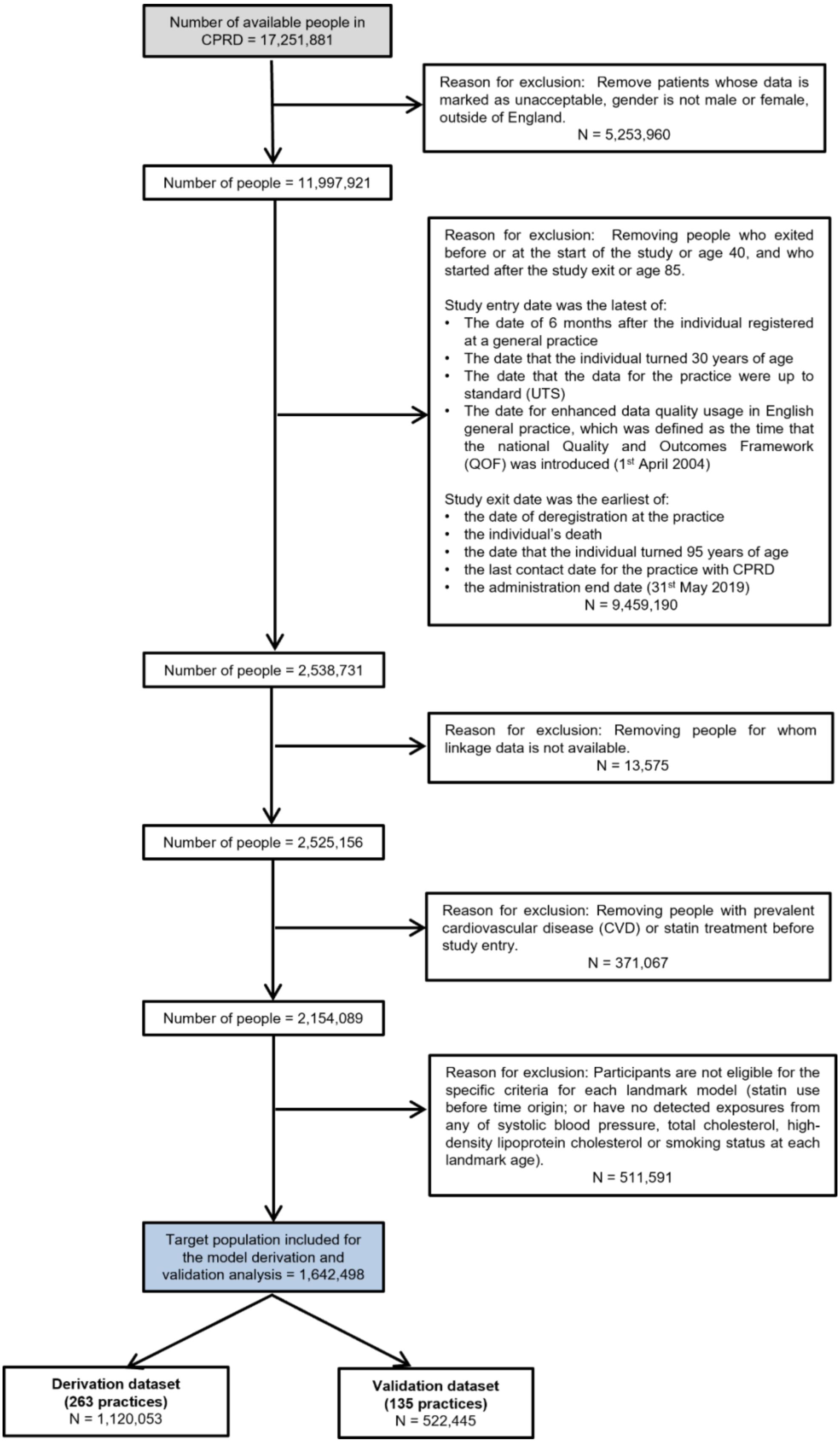

Fig 3. Number of participants and number of exposures at the start of each age among men in CPRD.

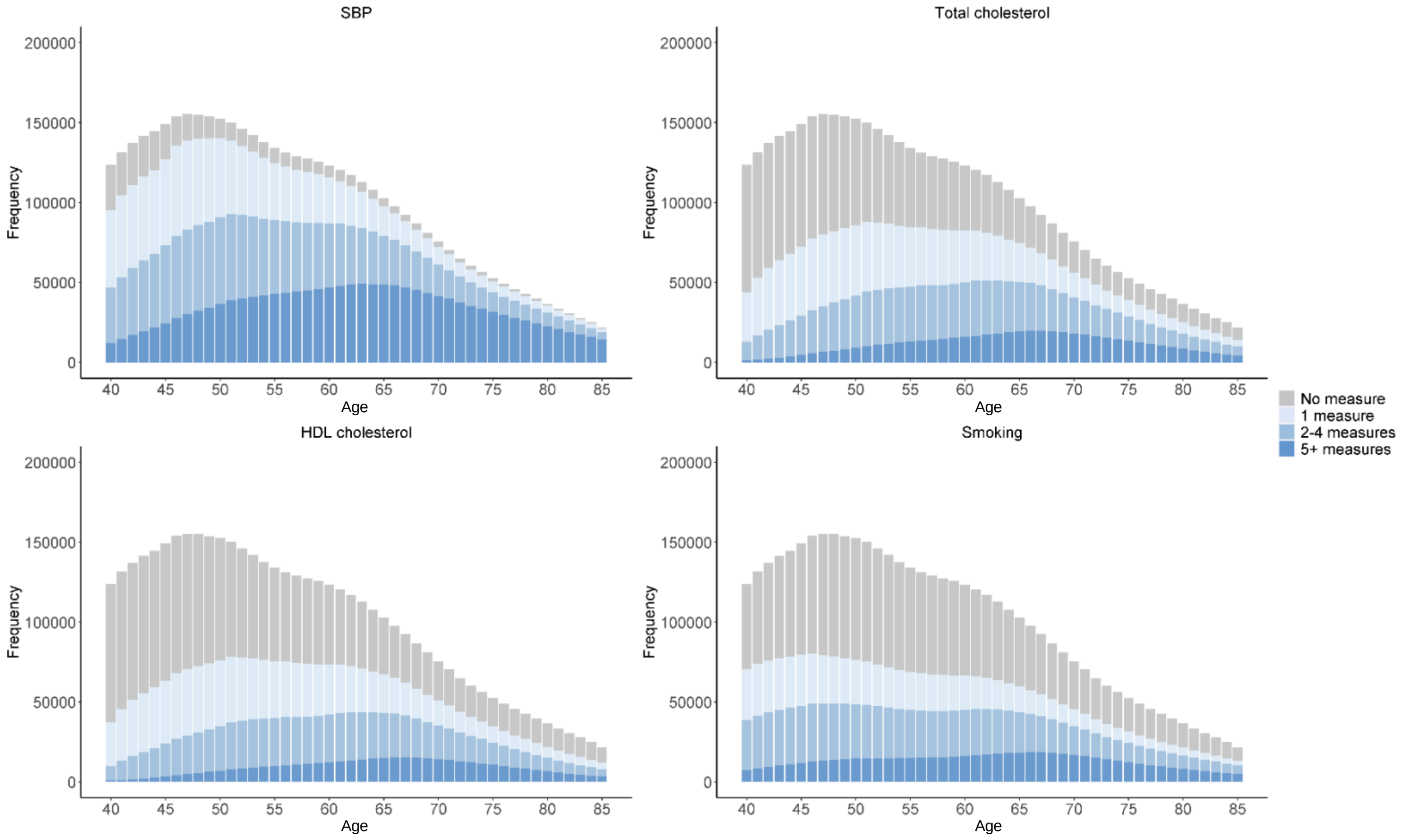

Fig 4. Number of participants and number of exposures at the start of each age among women in CPRD.

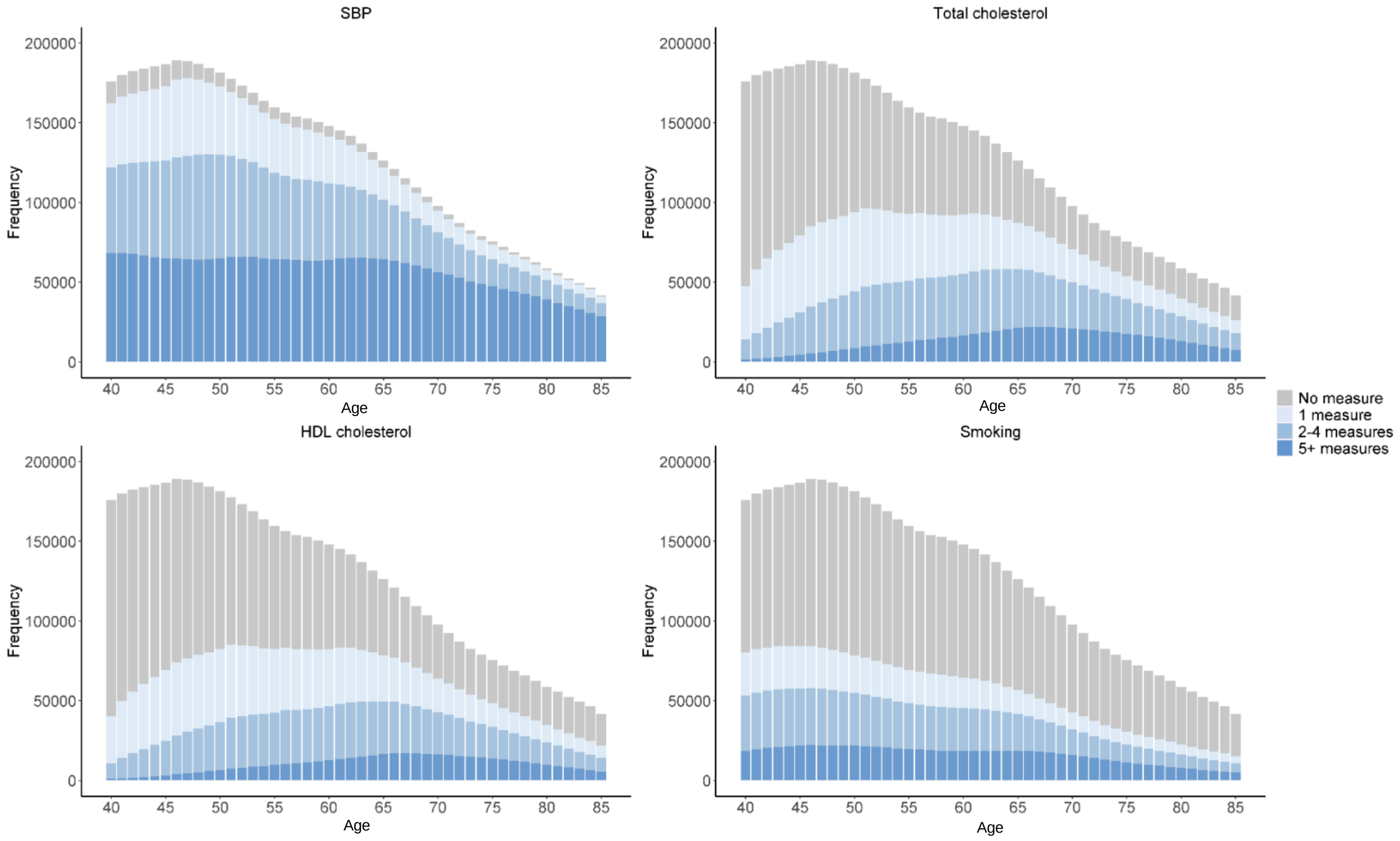

Fig 5. Venn diagram of incident cardiovascular events in the derivation dataset in CPRD.

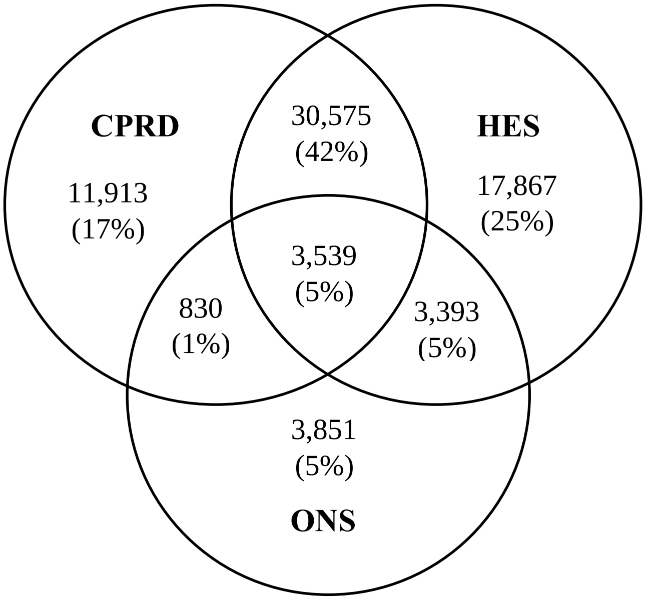

Events during follow-up in the derivation dataset recorded from primary care data in Clinical Practice Research Datalink (CPRD) (n=46,857 first events identified), secondary care data in Hospital Episode Statistics (HES) (n=55,374 first events identified), and mortality records in Office for National Statistics (ONS) (n=11,613 first events identified), England, United Kingdom, 2004-2019

Fig 6. Crude 10-year cardiovascular disease incidence rate by age and sex in the derivation dataset in CPRD.

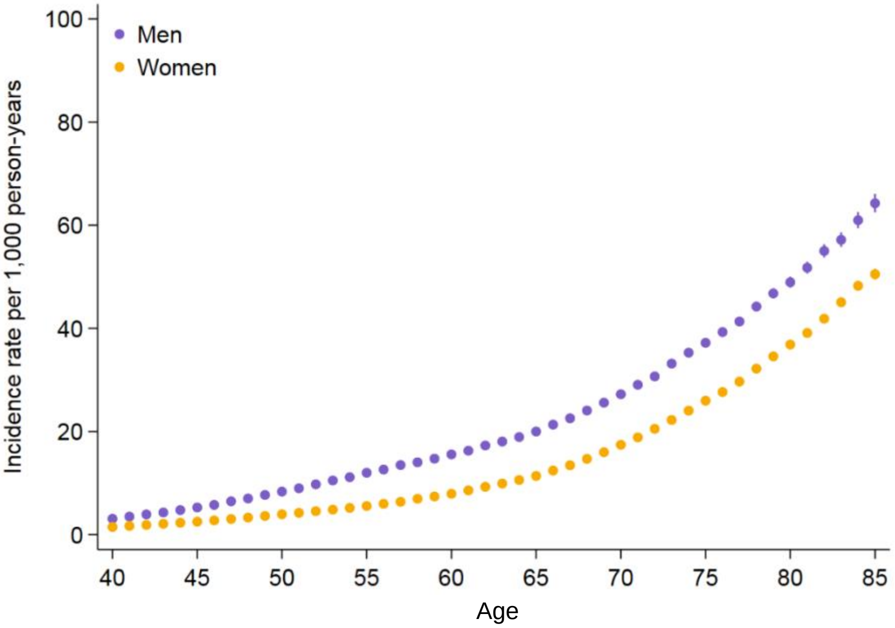

Fig 7. Mean level or percentage of risk factors by age in the derivation dataset in CPRD.

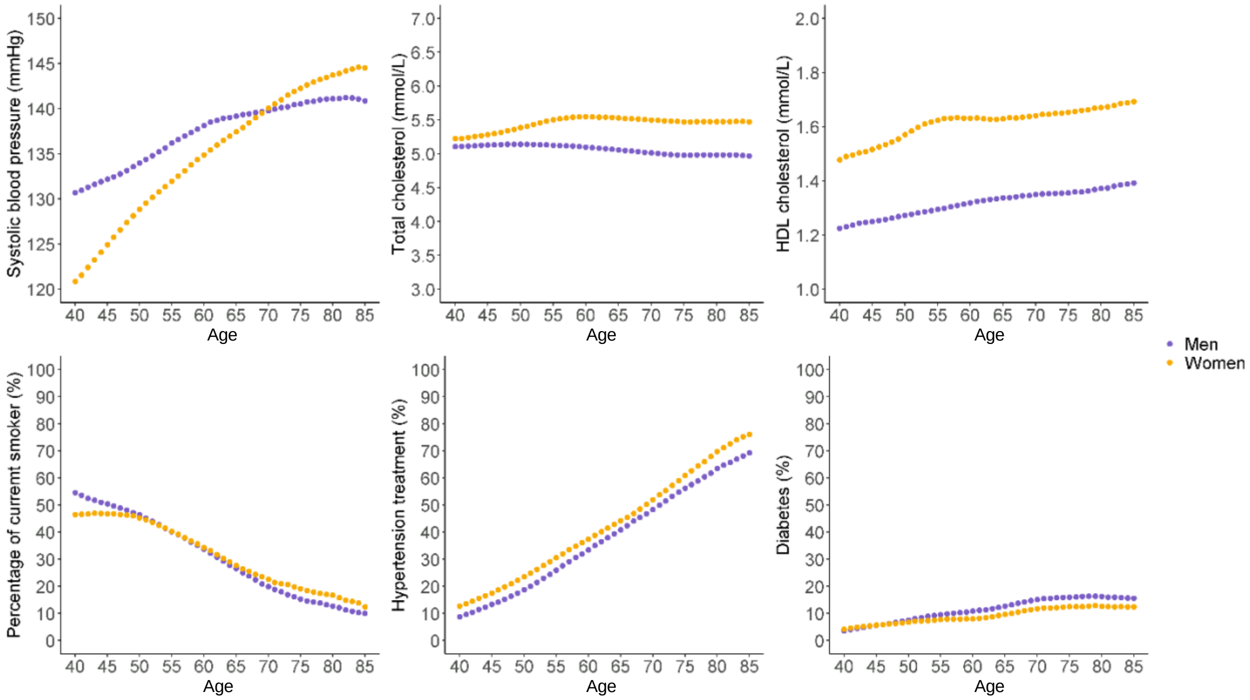

Fig 8. Histogram of predicted 10-year cardiovascular disease risk distributions by age group and sex in the validation dataset in CPRD.

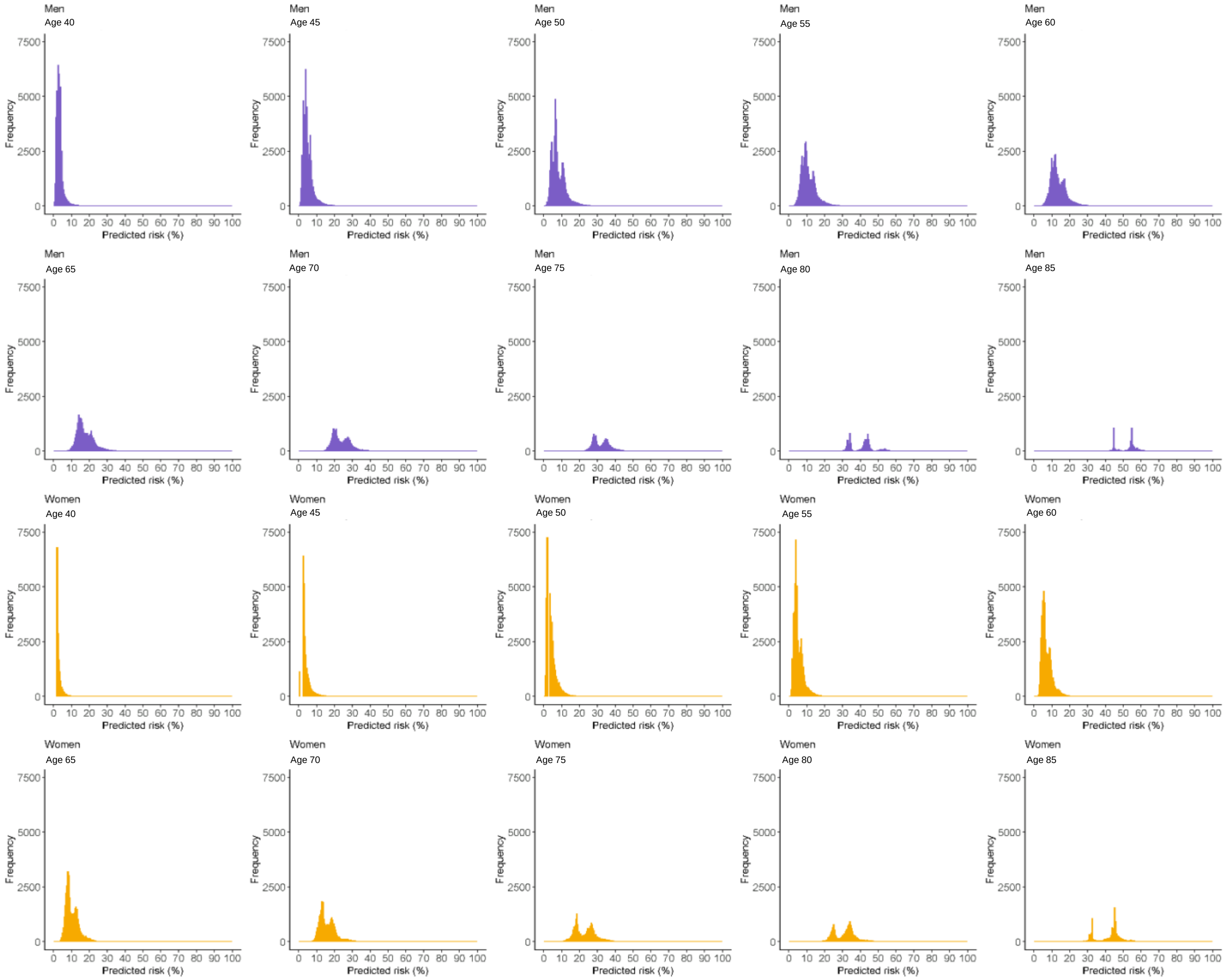

Fig 9. **Calibration plots by deciles of predicted risk at 5-year age groups among men in the validation dataset in CPRD.**

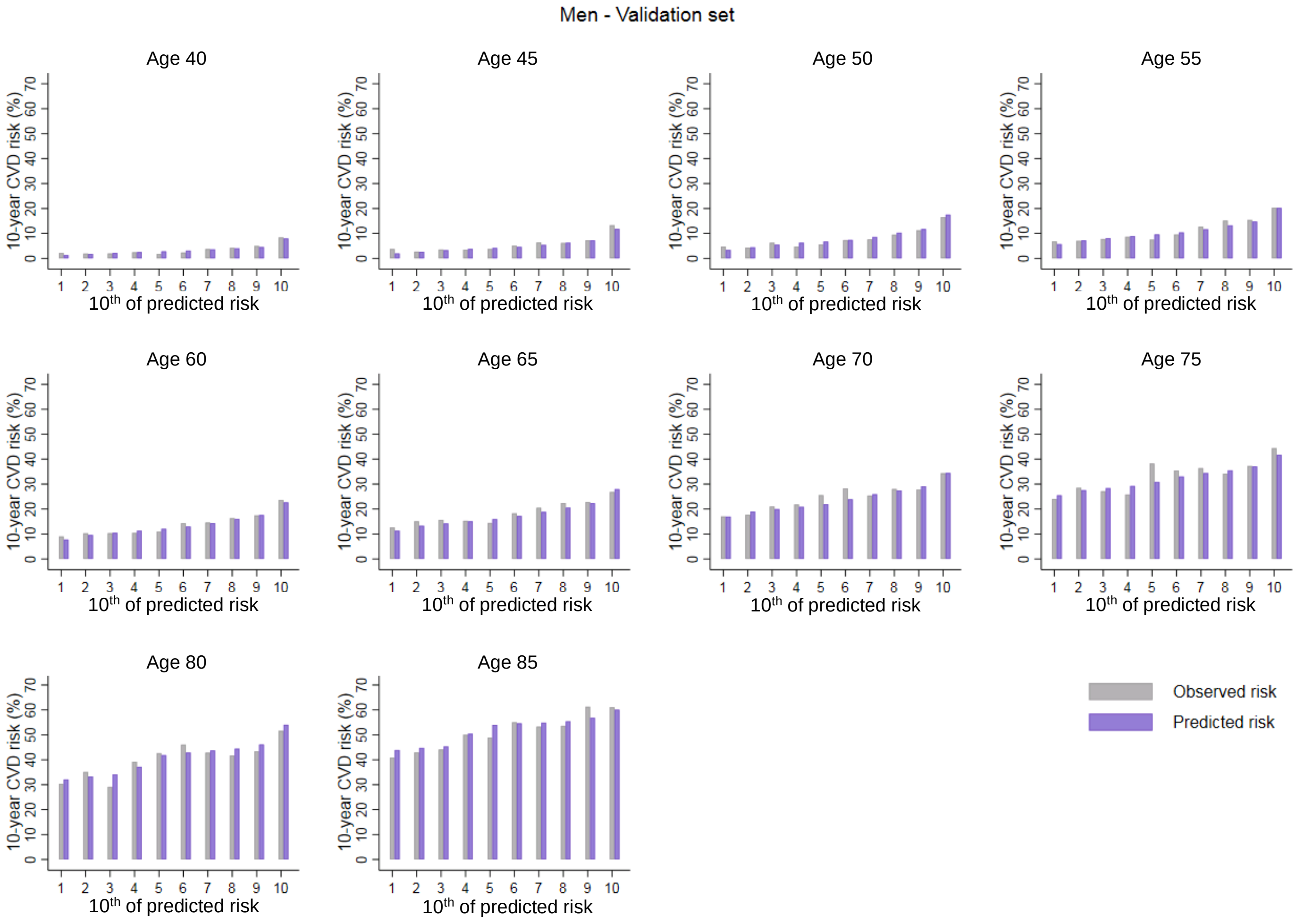

Fig 10. Calibration plots by deciles of predicted risk at 5-year age groups among women in the validation dataset in CPRD.

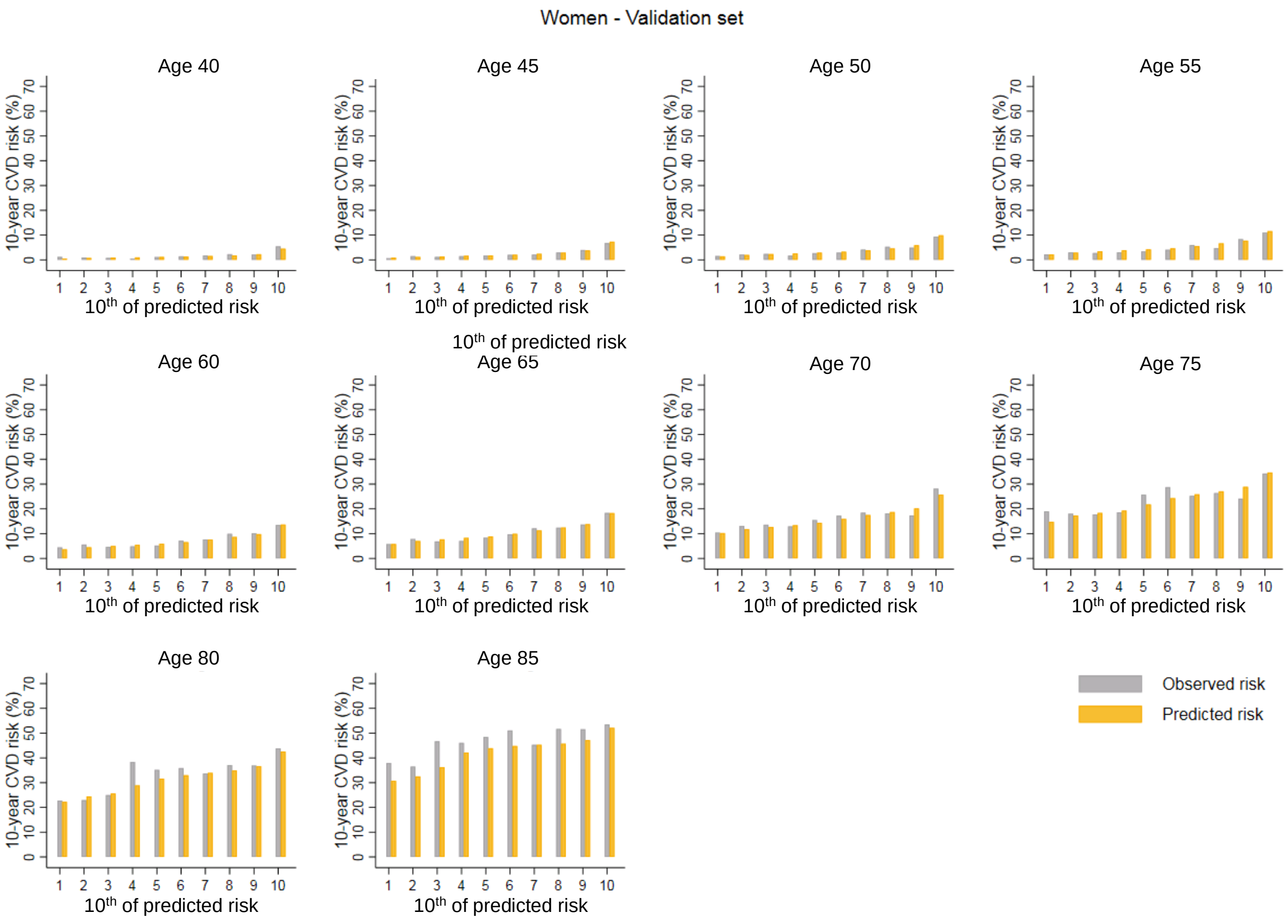

Fig 11. Calibration slopes by landmark age among men and women in the validation dataset in CPRD.

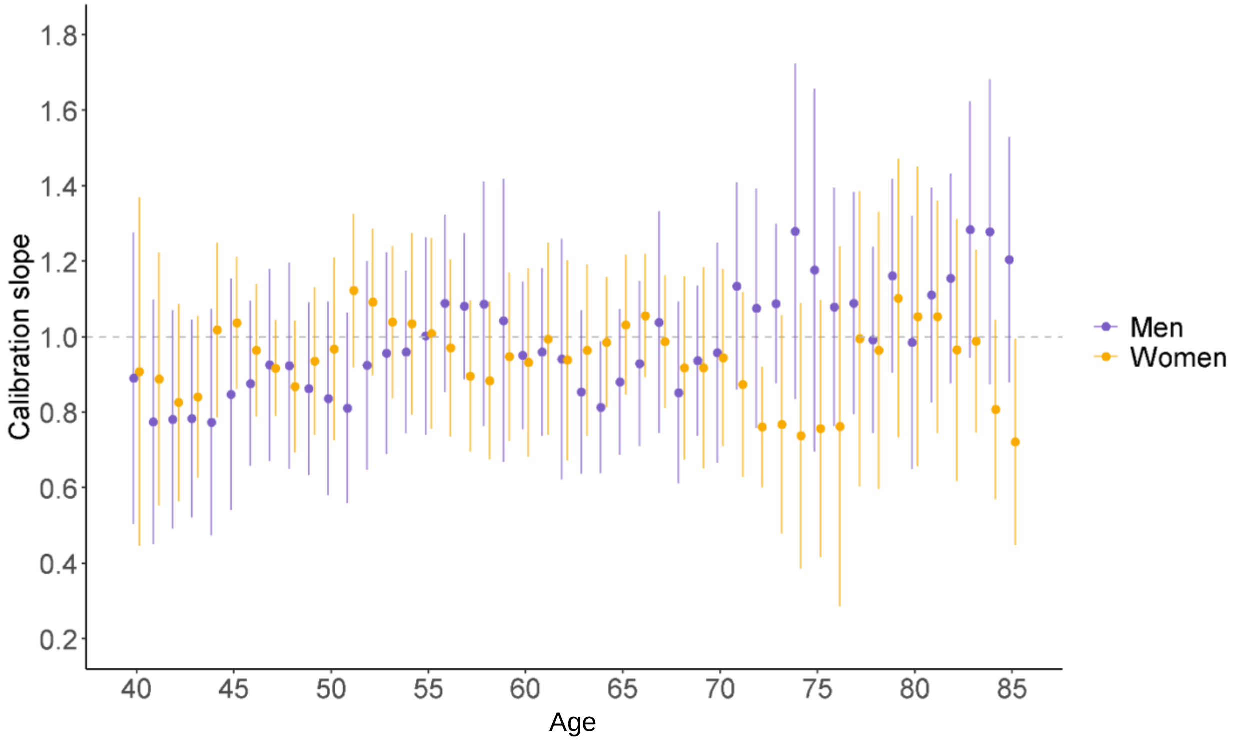

Fig 12. Flowchart showing selection of individuals for population health modelling in UK Biobank

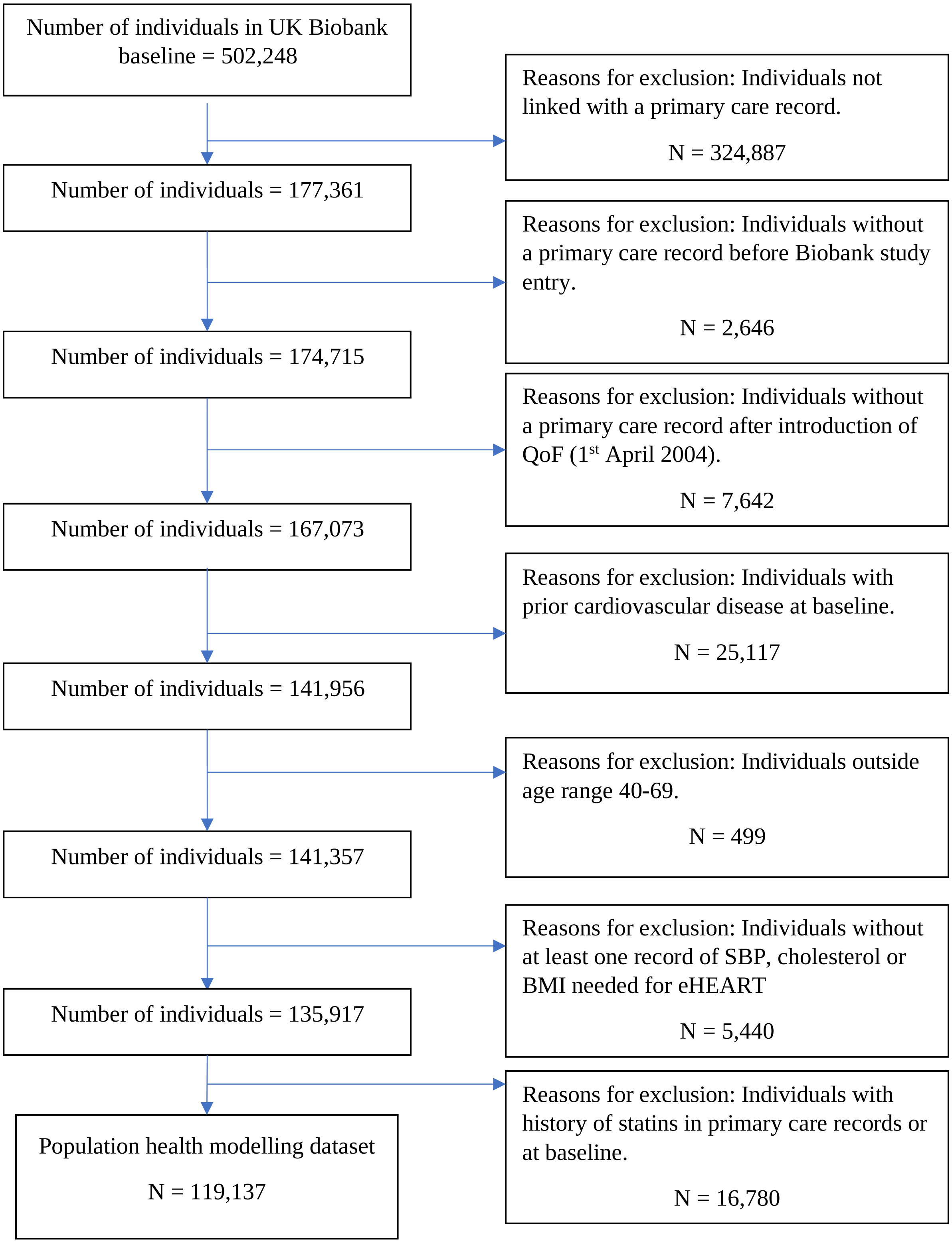

References

1. Paige E, Barrett J, Stevens D, Keogh RH, Sweeting MJ, Nazareth I, et al. Landmark Models for Optimizing the Use of Repeated Measurements of Risk Factors in Electronic Health Records to Predict Future Disease Risk. Am J Epidemiol [Internet]. 2018 Jul 1 [cited 2021 Nov 23];187(7):1530–8. Available from: https://academic.oup.com/aje/article/187/7/1530/4952104
