# Supplementary material for: Prioritising cardiovascular disease risk assessment to high risk individuals based on primary care records
Source: PLoS One. 2023 Sep 29;18(9):e0292240. doi: 10.1371/journal.pone.0292240 (PMC10540947; doi:10.1371/journal.pone.0292240)
Supplement: S2 File — (PDF) [file pone.0292240.s002.pdf]

Table 1. Code list of cardiovascular disease

Cardiovascular disease was defined as a combination of newly diagnoses of nonfatal or fatal events of coronary heart disease (CHD) (including myocardial infarction and angina), stroke, and transient ischemic attack (TIA), in line with the definition used in the QRISK3 CVD risk score[1]. In Clinical Practice Research Datalink (CPRD), diagnoses are coded using the hierarchical Read code system[1] and in the linked HES and ONS datasets, the International Classification of Disease 10th revision (ICD-10) codes were used[2].

Read code for CPRD data	
Read code	Description
G3...00	Ischaemic heart disease
G31..00	Arteriosclerotic heart disease
G32..00	Atherosclerotic heart disease
G33..00	IHD - Ischaemic heart disease
G30..00	Acute myocardial infarction
G301.00	Attack - heart
G302.00	Coronary thrombosis
G303.00	Cardiac rupture following myocardial infarction (MI)
G304.00	Heart attack
G305.00	MI - acute myocardial infarction
G306.00	Thrombosis - coronary
G307.00	Silent myocardial infarction
G309800	Coronary thrombosis
G309900	Myocardial Infarction
G300.00	Acute anterolateral infarction
G301.00	Other specified anterior myocardial infarction
G301000	Acute anteroapical infarction
G301100	Acute anteroseptal infarction
G301z00	Anterior myocardial infarction NOS
G302.00	Acute inferolateral infarction
G303.00	Acute inferoposterior infarction
G304.00	Posterior myocardial infarction NOS
G305.00	Lateral myocardial infarction NOS
G306.00	True posterior myocardial infarction
G307.00	Acute subendocardial infarction

G307000	Acute non-Q wave infarction
G307100	Acute non-ST segment elevation myocardial infarction
G308.00	Inferior myocardial infarction NOS
G309.00	Acute Q-wave infarct
G30A.00	Mural thrombosis
G30B.00	Acute posterolateral myocardial infarction
G30X.00	Acute transmural myocardial infarction of unspecif site
G30X000	Acute ST segment elevation myocardial infarction
G30y.00	Other acute myocardial infarction
G30y000	Acute atrial infarction
G30y100	Acute papillary muscle infarction
G30y200	Acute septal infarction
G30yz00	Other acute myocardial infarction NOS
G30z.00	Acute myocardial infarction NOS
G31..00	Other acute and subacute ischaemic heart disease
G319900	Acute/subacute IHD NOS
G310.00	Postmyocardial infarction syndrome
G310100	Dressler's syndrome
G311.00	Preinfarction syndrome
G311100	Crescendo angina
G311200	Impending infarction
G311300	Unstable angina
G311400	Angina at rest
G311000	Myocardial infarction aborted
G311010	MI - myocardial infarction aborted
G311100	Unstable angina
G311200	Angina at rest
G311300	Refractory angina
G311400	Worsening angina
G311500	Acute coronary syndrome
G311z00	Preinfarction syndrome NOS
G312.00	Coronary thrombosis not resulting in myocardial infarction

G31y.00	Other acute and subacute ischaemic heart disease
G31y000	Acute coronary insufficiency
G31y099	Acute coronary syndrome
G31y100	Microinfarction of heart
G31y200	Subendocardial ischaemia
G31y300	Transient myocardial ischaemia
G31yz00	Other acute and subacute ischaemic heart disease NOS
G32..00	Old myocardial infarction
G321.00	Healed myocardial infarction
G322.00	Personal history of myocardial infarction
G33..00	Angina pectoris
G330.00	Angina decubitus
G330000	Nocturnal angina
G330z00	Angina decubitus NOS
G331.00	Prinzmetal's angina
G331100	Variant angina pectoris
G332.00	Coronary artery spasm
G33z.00	Angina pectoris NOS
G33z000	Status anginosus
G33z100	Stenocardia
G33z200	Syncope anginosa
G33z300	Angina on effort
G33z400	Ischaemic chest pain
G33z500	Post infarct angina
G33z600	New onset angina
G33z700	Stable angina
G33zz00	Angina pectoris NOS
G34..00	Other chronic ischaemic heart disease
G349900	Chr. ischaemic heart dis. NOS
G340.00	Coronary atherosclerosis
G340100	Triple vessel disease of the heart
G340200	Coronary artery disease

G340000	Single coronary vessel disease
G340100	Double coronary vessel disease
G342.00	Atherosclerotic cardiovascular disease
G343.00	Ischaemic cardiomyopathy
G344.00	Silent myocardial ischaemia
G34y.00	Other specified chronic ischaemic heart disease
G34y000	Chronic coronary insufficiency
G34y100	Chronic myocardial ischaemia
G34yz00	Other specified chronic ischaemic heart disease NOS
G34z.00	Other chronic ischaemic heart disease NOS
G34z000	Asymptomatic coronary heart disease
G35..00	Subsequent myocardial infarction
G350.00	Subsequent myocardial infarction of anterior wall
G351.00	Subsequent myocardial infarction of inferior wall
G353.00	Subsequent myocardial infarction of other sites
G35X.00	Subsequent myocardial infarction of unspecified site
G36..00	Certain current complication follow acute myocardial infarct
G360.00	Haemopericardium/current comp follow acute myocardial infarct
G361.00	Atrial septal defect/curr comp follow acute myocardial infarct
G362.00	Ventricular septal defect/curr comp follow acute myocardial infarction
G363.00	Ruptur cardiac wall w'out haemopericard/cur comp follow ac MI
G364.00	Ruptur chordae tendinae/curr comp follow acute myocardial infarct
G365.00	Rupture papillary muscle/curr comp follow acute myocardial infarct
G366.00	Thrombosis atrium, auric append&vent/curr comp follow acute MI
G38..00	Postoperative myocardial infarction
G380.00	Postoperative transmural myocardial infarction anterior wall
G381.00	Postoperative transmural myocardial infarction inferior wall
G382.00	Postoperative transmural myocardial infarction other sites
G383.00	Postoperative transmural myocardial infarction unspec site
G384.00	Postoperative subendocardial myocardial infarction
G38z.00	Postoperative myocardial infarction, unspecified
G3y..00	Other specified ischaemic heart disease

G3z..00	Ischaemic heart disease NOS
G501.00	Post infarction pericarditis
Gyu3400	[X]Acute transmural myocardial infarction of unspecif site
F423600	Amaurosis fugax
Fyu5500	[X]Other transnt cerebral ischaemic attacks+related syndromes
G63y000	Cerebral infarct due to thrombosis of precerebral arteries
G63y100	Cerebral infarction due to embolism of precerebral arteries
G64..00	Cerebral arterial occlusion
G641.00	CVA - cerebral artery occlusion
G642.00	Infarction - cerebral
G643.00	Stroke due to cerebral arterial occlusion
G640.00	Cerebral thrombosis
G640000	Cerebral infarction due to thrombosis of cerebral arteries
G641.00	Cerebral embolism
G641100	Cerebral embolus
G641000	Cerebral infarction due to embolism of cerebral arteries
G64z.00	Cerebral infarction NOS
G64z100	Brainstem infarction NOS
G64z200	Cerebellar infarction
G64z990	Cerebral A. occlusion NOS
G64z000	Brainstem infarction
G64z100	Wallenberg syndrome
G64z110	Lateral medullary syndrome
G64z200	Left sided cerebral infarction
G64z300	Right sided cerebral infarction
G64z400	Infarction of basal ganglia
G65..00	Transient cerebral ischaemia
G651.00	Drop attack
G652.00	Transient ischaemic attack
G653.00	Vertebro-basilar insufficiency
G659900	Transient Ischaemic Attacks
G650.00	Basilar artery syndrome

G650100	Insufficiency - basilar artery
G652.00	Subclavian steal syndrome
G653.00	Carotid artery syndrome hemispheric
G654.00	Multiple and bilateral precerebral artery syndromes
G656.00	Vertebrobasilar insufficiency
G65y.00	Other transient cerebral ischaemia
G65z.00	Transient cerebral ischaemia NOS
G65z990	Transient Ischaemic Attacks
G65z000	Impending cerebral ischaemia
G65z100	Intermittent cerebral ischaemia
G65zz00	Transient cerebral ischaemia NOS
G66..00	Stroke and cerebrovascular accident unspecified
G661.00	CVA unspecified
G662.00	Stroke unspecified
G663.00	CVA - Cerebrovascular accident unspecified
G669800	Stroke/CVA - undefined
G669900	Stroke
G667.00	Left sided CVA
G668.00	Right sided CVA
G676000	Cereb infarct due cerebral venous thrombosis, nonpyogenic
G6W..00	Cereb infarct due unspcf occlus/stenos precerebr arteries
G6X..00	Cerebrl infarctn due/unspcf occlusn or sten/cerebrl artrs
Gyu6300	[X]Cerebrl infarctn due/unspcf occlusn or sten/cerebrl artrs
Gyu6400	[X]Other cerebral infarction
Gyu6500	[X]Occlusion and stenosis of other precerebral arteries
Gyu6600	[X]Occlusion and stenosis of other cerebral arteries
ZV12D00	[V]Personal history of transient ischaemic attack

ICD10 code for HES and ONS data	
ICD10-code	description
G45	transient ischaemic attack and related syndromes

G45.0	transient ischaemic attack and related syndromes
G45.1	transient ischaemic attack and related syndromes
G45.2	transient ischaemic attack and related syndromes
G45.3	transient ischaemic attack and related syndromes
G45.4	transient ischaemic attack and related syndromes
G45.8	transient ischaemic attack and related syndromes
G45.9	transient ischaemic attack and related syndromes
I20	angina pectoris
I20.0	angina pectoris
I20.1	angina pectoris
I20.8	angina pectoris
I20.9	angina pectoris
I21	acute myocardial infarction
I21.0	acute myocardial infarction
I21.1	acute myocardial infarction
I21.2	acute myocardial infarction
I21.3	acute myocardial infarction
I21.4	acute myocardial infarction
I21.9	acute myocardial infarction
I22	subsequent myocardial infarction
I22.0	subsequent myocardial infarction
I22.1	subsequent myocardial infarction
I22.8	subsequent myocardial infarction
I22.9	subsequent myocardial infarction
I23	complications after myocardial infarction
I23.0	complications after myocardial infarction
I23.1	complications after myocardial infarction
I23.2	complications after myocardial infarction
I23.3	complications after myocardial infarction
I23.4	complications after myocardial infarction
I23.5	complications after myocardial infarction
I23.6	complications after myocardial infarction

I23.8	complications after myocardial infarction
I24	other acute ischaemic heart disease
I24.0	other acute ischaemic heart disease
I24.1	other acute ischaemic heart disease
I24.8	other acute ischaemic heart disease
I24.9	other acute ischaemic heart disease
I25	chronic ischaemic heart disease
I25.0	chronic ischaemic heart disease
I25.1	chronic ischaemic heart disease
I25.2	chronic ischaemic heart disease
I25.3	chronic ischaemic heart disease
I25.4	chronic ischaemic heart disease
I25.5	chronic ischaemic heart disease
I25.6	chronic ischaemic heart disease
I25.8	chronic ischaemic heart disease
I25.9	chronic ischaemic heart disease
I63	cerebral infarction
I63.0	cerebral infarction
I63.1	cerebral infarction
I63.2	cerebral infarction
I63.3	cerebral infarction
I63.4	cerebral infarction
I63.5	cerebral infarction
I63.6	cerebral infarction
I63.8	cerebral infarction
I63.9	cerebral infarction
I64	stroke not specified as haemorrhage or infarction

Table 2. The RECORD statement[17]– checklist of items, extended from the STROBE statement, that should be reported in observational studies using routinely collected health data.

	Item No.	STROBE items	Location in manuscript where items are reported	RECORD items	Location in manuscript where items are reported
**Title and abstract**					
	1	(a) Indicate the study's design with a commonly used term in the title or the abstract (b) Provide in the abstract an informative and balanced summary of what was done and what was found	3	RECORD 1.1: The type of data used should be specified in the title or abstract. When possible, the name of the databases used should be included.<br><br>RECORD 1.2: If applicable, the geographic region and timeframe within which the study took place should be reported in the title or abstract.<br><br>RECORD 1.3: If linkage between databases was conducted for the study, this should be clearly stated in the title or abstract.	3<br><br>3<br><br>3
**Introduction**					
Background rationale	2	Explain the scientific background and rationale for the investigation being reported	5		
Objectives	3	State specific objectives, including any prespecified hypotheses	5		
**Methods**					
Study Design	4	Present key elements of study design early in the paper	6		
Setting	5	Describe the setting, locations, and relevant dates, including periods of recruitment, exposure, follow-up, and data collection	6		
Participants	6	*(a) Cohort study* - Give the eligibility criteria, and the sources and methods of selection of participants. Describe methods of follow-up<br>*Case-control study* - Give the eligibility criteria, and	6	RECORD 6.1: The methods of study population selection (such as codes or algorithms used to identify subjects) should be listed in detail. If this is not possible, an	6

		the sources and methods of case ascertainment and control selection. Give the rationale for the choice of cases and controls<br>*Cross-sectional study* - Give the eligibility criteria, and the sources and methods of selection of participants<br><br>*(b) Cohort study* - For matched studies, give matching criteria and number of exposed and unexposed<br>*Case-control study* - For matched studies, give matching criteria and the number of controls per case		explanation should be provided.<br><br>RECORD 6.2: Any validation studies of the codes or algorithms used to select the population should be referenced. If validation was conducted for this study and not published elsewhere, detailed methods and results should be provided.<br><br>RECORD 6.3: If the study involved linkage of databases, consider use of a flow diagram or other graphical display to demonstrate the data linkage process, including the number of individuals with linked data at each stage.	 <br>6<br><br><br><br><br><br><br><br><br><br>eFigure2
Variables	7	Clearly define all outcomes, exposures, predictors, potential confounders, and effect modifiers. Give diagnostic criteria, if applicable.	7	RECORD 7.1: A complete list of codes and algorithms used to classify exposures, outcomes, confounders, and effect modifiers should be provided. If these cannot be reported, an explanation should be provided.	7, Web appendix 1
Data sources/ measurement	8	For each variable of interest, give sources of data and details of methods of assessment (measurement).<br>Describe comparability of assessment methods if there is more than one group	7		
Bias	9	Describe any efforts to address potential sources of bias	7-8		
Study size	10	Explain how the study size was arrived at	eFigure2, eFigure 12		
Quantitative variables	11	Explain how quantitative variables were handled in the analyses. If applicable, describe which groupings were chosen, and why	7		
Statistical methods	12	(a) Describe all statistical methods, including those	7-10		

		used to control for confounding<br>(b) Describe any methods used to examine subgroups and interactions<br>(c) Explain how missing data were addressed<br>(d) *Cohort study* - If applicable, explain how loss to follow-up was addressed<br>*Case-control study* - If applicable, explain how matching of cases and controls was addressed<br>*Cross-sectional study* - If applicable, describe analytical methods taking account of sampling strategy<br>(e) Describe any sensitivity analyses			
Data access and cleaning methods		N/A		RECORD 12.1: Authors should describe the extent to which the investigators had access to the database population used to create the study population.<br><br>RECORD 12.2: Authors should provide information on the data cleaning methods used in the study.	6<br><br>6, eFigure2
Linkage		N/A		RECORD 12.3: State whether the study included person-level, institutional-level, or other data linkage across two or more databases. The methods of linkage and methods of linkage quality evaluation should be provided.	6
**Results**					
Participants	13	(a) Report the numbers of individuals at each stage of the study (*e.g.*, numbers potentially eligible, examined for eligibility, confirmed eligible, included in the study, completing follow-up, and analysed)<br>(b) Give reasons for non-participation at each stage.	11, eFigure2, eFigure12	RECORD 13.1: Describe in detail the selection of the persons included in the study (*i.e.*, study population selection) including filtering based on data quality, data availability and linkage. The selection of included persons can be described in the text and/or by	6, eFigure2, eFigure12

		(c) Consider use of a flow diagram		means of the study flow diagram.	
Descriptive data	14	(a) Give characteristics of study participants (*e.g.*, demographic, clinical, social) and information on exposures and potential confounders<br>(b) Indicate the number of participants with missing data for each variable of interest<br>(c) *Cohort study* - summarise follow-up time (*e.g.*, average and total amount)	Table 1		
Outcome data	15	*Cohort study* - Report numbers of outcome events or summary measures over time<br>*Case-control study* - Report numbers in each exposure category, or summary measures of exposure<br>*Cross-sectional study* - Report numbers of outcome events or summary measures	eFigure 5, eFigure 6		
Main results	16	(a) Give unadjusted estimates and, if applicable, confounder-adjusted estimates and their precision (e.g., 95% confidence interval). Make clear which confounders were adjusted for and why they were included<br>(b) Report category boundaries when continuous variables were categorized<br>(c) If relevant, consider translating estimates of relative risk into absolute risk for a meaningful time period	eTable3		
Other analyses	17	Report other analyses done—e.g., analyses of subgroups and interactions, and sensitivity analyses	13-15 Table 2, eTable 6		
**Discussion**					
Key results	18	Summarise key results with reference to study objectives	15-17		
Limitations	19	Discuss limitations of the study, taking into account	17	RECORD 19.1: Discuss the implications of using	17

		sources of potential bias or imprecision. Discuss both direction and magnitude of any potential bias		data that were not created or collected to answer the specific research question(s). Include discussion of misclassification bias, unmeasured confounding, missing data, and changing eligibility over time, as they pertain to the study being reported.	
Interpretation	20	Give a cautious overall interpretation of results considering objectives, limitations, multiplicity of analyses, results from similar studies, and other relevant evidence	17		
Generalisability	21	Discuss the generalisability (external validity) of the study results	16-18		
**Other Information**					
Funding	22	Give the source of funding and the role of the funders for the present study and, if applicable, for the original study on which the present article is based	19		
Accessibility of protocol, raw data, and programming code		N/A		RECORD 22.1: Authors should provide information on how to access any supplemental information such as the study protocol, raw data, or programming code.	19

Table 3. TRIPOD[18] Checklist: Prediction Model Development and Validation.

Section/Topic			Checklist Item	Page
**Title and abstract**				
Title	1	D;V	Identify the study as developing and/or validating a multivariable prediction model, the target population, and the outcome to be predicted.	1
Abstract	2	D;V	Provide a summary of objectives, study design, setting, participants, sample size, predictors, outcome, statistical analysis, results, and conclusions.	3
**Introduction**				
Background and objectives	3a	D;V	Explain the medical context (including whether diagnostic or prognostic) and rationale for developing or validating the multivariable prediction model, including references to existing models.	5
	3b	D;V	Specify the objectives, including whether the study describes the development or validation of the model or both.	5
**Method**				
Source of data	4a	D;V	Describe the study design or source of data (e.g., randomized trial, cohort, or registry data), separately for the development and validation data sets, if applicable.	6
	4b	D;V	Specify the key study dates, including start of accrual; end of accrual; and, if applicable, end of follow-up.	6
Participants	5a	D;V	Specify key elements of the study setting (e.g., primary care, secondary care, general population) including number and location of centres.	6
	5b	D;V	Describe eligibility criteria for participants.	6
	5c	D;V	Give details of treatments received, if relevant.	-
Outcome	6a	D;V	Clearly define the outcome that is predicted by the prediction model, including how and when assessed.	7
	6b	D;V	Report any actions to blind assessment of the outcome to be predicted.	7
Predictors	7a	D;V	Clearly define all predictors used in developing or validating the multivariable prediction model, including how and when they were measured.	7
	7b	D;V	Report any actions to blind assessment of predictors for the outcome and other predictors.	7
Sample size	8	D;V	Explain how the study size was arrived at.	6
Missing data	9	D;V	Describe how missing data were handled (e.g., complete-case analysis, single imputation, multiple imputation) with details of any imputation method.	7-8
Statistical analysis methods	10a	D	Describe how predictors were handled in the analyses.	7
	10b	D	Specify type of model, all model-building procedures (including any predictor selection), and method for internal validation.	7-8
	10c	V	For validation, describe how the predictions were calculated.	8-10
	10d	D;V	Specify all measures used to assess model performance and, if relevant, to compare multiple models.	8-10

	10e	V	Describe any model updating (e.g., recalibration) arising from the validation, if done.	9
Risk groups	11	D;V	Provide details on how risk groups were created, if done.	10
Development vs. validation	12	V	For validation, identify any differences from the development data in setting, eligibility criteria, outcome, and predictors.	10
**Results**				
Participants	13a	D;V	Describe the flow of participants through the study, including the number of participants with and without the outcome and, if applicable, a summary of the follow-up time. A diagram may be helpful.	11-12
	13b	D;V	Describe the characteristics of the participants (basic demographics, clinical features, available predictors), including the number of participants with missing data for predictors and outcome.	11
	13c	V	For validation, show a comparison with the development data of the distribution of important variables (demographics, predictors and outcome).	12
Model development	14a	D	Specify the number of participants and outcome events in each analysis.	11
	14b	D	If done, report the unadjusted association between each candidate predictor and outcome.	-
Model specification	15a	D	Present the full prediction model to allow predictions for individuals (i.e., all regression coefficients, and model intercept or baseline survival at a given time point).	12
	15b	D	Explain how to the use the prediction model.	13
Model performance	16	D;V	Report performance measures (with CIs) for the prediction model.	12
Model-updating	17	V	If done, report the results from any model updating (i.e., model specification, model performance).	-
**Discussion**				
Limitations	18	D;V	Discuss any limitations of the study (such as nonrepresentative sample, few events per predictor, missing data).	17-18
Interpretation	19a	V	For validation, discuss the results with reference to performance in the development data, and any other validation data.	15-16
	19b	D;V	Give an overall interpretation of the results, considering objectives, limitations, results from similar studies, and other relevant evidence.	16-18
Implications	20	D;V	Discuss the potential clinical use of the model and implications for future research.	18
**Other information**				
Supplementary information	21	D;V	Provide information about the availability of supplementary resources, such as study protocol, Web calculator, and data sets.	20
Funding	22	D;V	Give the source of funding and the role of the funders for the present study.	19

Text 1. Two-stage dynamic landmark age model for risk prediction

Dynamic landmark age modelling

To optimize the use of repeated measurements of risk factors in electronic health records to predict future CVD risk, we used sliding landmark approach as described in our previous study[3] to construct 10-year CVD risk prediction models. The schematic of landmark age approach is presented in Web Figure 2. A landmark age is a reference point at which we use risk factor values collected prior to that age and from which to predict future risk[3]. In the derivation dataset, we derived a series of ninety two age- and sex-specific predictions models (i.e., for men and women and at ages 40, 41, 42, ...,85, denoted as "landmark ages"). Participants contributed to the models if they have 1) registered with a general practice at the landmark age, 2) no CVD diagnoses prior to the landmark age, and 3) no statin prescription prior to the landmark age.

We selected the key cardiovascular risk factors as those used in the validated 2013 ACC/AHA Pooled Cohort Equations[4]: age, sex, total cholesterol, high-density lipoprotein (HDL) cholesterol, systolic blood pressure (SBP), use of antihypertensive therapy, diabetes mellitus status, and smoking status. Values of SBP, total cholesterol and HDL cholesterol were standardised by centering on sex- specific means and dividing by the standard deviation (using means and standard deviations calculated from the first measurement from each individual). Age and sex were known for all participants. Values for diabetes mellitus status, anti-hypertensive therapy usage and statin therapy usage were set to zero until the first available health record indicated otherwise (i.e. for diabetes mellitus: at least one diabetes diagnostic code [Read code or diabetes test] plus either an additional diagnostic code or diabetes drug prescription[5]; first prescription of a blood-pressure or cholesterol medication) from which time the values were set to one. Repeat measurements of smoking status, systolic blood pressure, total cholesterol and HDL cholesterol were first summarised using age- and sex-specific multivariate mixed models[6] and entered the prediction model as single summary measures as described below.

The landmark age approach comprises of two stages:

Stage 1: *Summarising repeated measures of risk factors using multivariate mixed- effects linear regression models*

Let $Smoking\_status_{ij}$, SBP_{ij} , $Total\_cholesterol_{ij}$, $HDL\_cholesterol_{ij}$, BP_med_{ij} and $Statin_{ij}$ denote all the repeat measurements of smoking status, SBP, total cholesterol, HDL cholesterol, indication of blood pressure-lowering medication, and indication of statin initiation for individual i recorded at

measurement j. For males and females separately, for each landmark age La = 40, 41, 42, ...,85, we fit a multivariate mixed-effect model with a correlated covariance structure:

$$SBP_{ij} = \alpha_1 + \beta_1 * t_{ij} + \gamma * BP\_med_{ij} + u_{1i} + \varepsilon_{1ij}$$

$$Total\_cholesterol_{ij} = \alpha_2 + \beta_2 * t_{ij} + \delta * Statin_{ij} + u_{2i} + \varepsilon_{2ij}$$

$$HDL\ cholesterol_{ij} = \alpha_3 + \beta_3 * t_{ij} + u_{3i} + \varepsilon_{3ij}$$

$$Smoking\_status_{ij} = \alpha_4 + \beta_4 * t_{ij} + u_{4i} + \varepsilon_{4ij}$$

Where $$\begin{bmatrix} u_{1i} \\ u_{2i} \\ u_{3i} \\ u_{4i} \end{bmatrix} \sim multivariate\ normal \left(\begin{bmatrix} 0 \\ 0 \\ 0 \\ 0 \end{bmatrix}, \begin{bmatrix} \sigma_1^2 & \sigma_{12} & \sigma_{13} & \sigma_{14} \\ \sigma_{12} & \sigma_2^2 & \sigma_{23} & \sigma_{24} \\ \sigma_{13} & \sigma_{23} & \sigma_3^2 & \sigma_{34} \\ \sigma_{14} & \sigma_{24} & \sigma_{34} & \sigma_4^2 \end{bmatrix} \right)$$

And $$\begin{bmatrix} \varepsilon_{1ij} \\ \varepsilon_{2ij} \\ \varepsilon_{3ij} \\ \varepsilon_{4ij} \end{bmatrix} \sim multivariate\ normal \left(\begin{bmatrix} 0 \\ 0 \\ 0 \\ 0 \end{bmatrix}, \begin{bmatrix} \sigma_{e1}^2 & 0 & 0 & 0 \\ 0 & \sigma_{e2}^2 & 0 & 0 \\ 0 & 0 & \sigma_{e3}^2 & 0 \\ 0 & 0 & 0 & \sigma_{e4}^2 \end{bmatrix} \right)$$

Here α_1, α_2, α_3, α_4 represent fixed intercepts for each risk factor, β_1, β_2, β_3, $\beta_4$represent fixed slopes for each risk factor, γ represents an adjustment factor in systolic blood pressure levels for those with an indication of blood pressure-lowering medication and δ represents an adjustment factor in total cholesterol for those with an indication of statin medication.

Terms u_{1i}, u_{2i}, u_{3i} and u_{4i} represent random intercepts for each risk factor and are correlated between risk factors. These random intercepts are interpreted as the difference in the average level of the predictor for this individual compared to the population average level.

Finally, ε_{1ij} , ε_{2ij} , ε_{3ij} and ε_{4ij} represent uncorrelated residual errors for each risk factor.

This model allows incomplete records of the risk factors and includes all individuals with at least one measurement from at least one risk factor (see Web Figure 3). The correlation structure between the risk factors is estimated from individuals with observed data on more than one risk factor. Thus, the model assumes that, for each landmark age, risk factor values from individuals with incomplete data are from the same multivariate normal distribution for risk factor values for individuals with observed data (that is, assuming “missing at random”).

Our model assumes that all risk factors jointly follow a multivariate normal distribution, which is plausible for SBP, total cholesterol, HDL cholesterol but less plausible for smoking status which is defined as a binary variable (yes for current/ever smoker; no for never smoker). However, inference based from the multivariate normal distribution may often be reasonable even if the multivariate normality does not hold, especially in the context of imputation of missing data[7] and regression calibration[8,9].

In our previous work[3], we restricted the model derivation to repeat measurements recorded **before** the landmark age, i.e. $j \leq La$. However, we found slight improvements in sensitivity analyses when we used all available repeated measurements recorded **before** and **after** the landmark age, due to extra precision on parameter estimates. We accept a limitation is that it ignores informative censoring of individuals due to death or CVD events, however, our previous work shows informative censoring has little effect on the long-term usual levels of the included risk factors.

Best linear unbiased predictors (BLUPS)[10] are estimated for each risk factor for the random intercepts u_{1i}, u_{2i}, u_{3i} and u_{4i} using observed data for $j \leq La$ [11]. Note the restriction to only repeat measurements before the landmark age is important here, as the prediction model is intended for use in clinical practice where only past data will be available. The BLUPs are estimated as the mean of the empirical Bayes posterior distribution of the random intercepts conditional on observed risk factor measurements. Using the properties of multivariate normal distributions, this is also a multivariate normal distribution, and an exact formula for the mean can be calculated[12].

Specifically, for individual i

$$\begin{bmatrix} \hat{u}_{1i} \\ \hat{u}_{2i} \\ \hat{u}_{3i} \\ \hat{u}_{4i} \end{bmatrix} = GZ^T(ZGZ^T + \Sigma)^{-1}(Y - X\beta)$$

Here Y is the vector of risk factor observations, G is the covariance matrix of the random

effects = $\begin{bmatrix} \sigma_1^2 & \sigma_{12} & \sigma_{13} & \sigma_{14} \\ \sigma_{12} & \sigma_2^2 & \sigma_{23} & \sigma_{24} \\ \sigma_{13} & \sigma_{23} & \sigma_3^2 & \sigma_{34} \\ \sigma_{14} & \sigma_{24} & \sigma_{34} & \sigma_4^2 \end{bmatrix}$, Z is the design matrix which selects the corresponding random effect for each risk factor, Z^T is the matrix transpose of Z and $\sum$ is a diagonal matrix containing the corresponding residual variance for each risk factor. Importantly, due to the correlations structure between the random

intercepts, BLUPS can be estimated for all individuals with at least one repeat measurement for at least one risk factor.

Stage 2: *Estimating 10-year CVD risk using landmark age- and sex-specific Weibull proportional hazards models, accounting for future statin initiation effect.*

In the second stage, ten-year CVD risk was modelled using landmark age- and sex- specific Weibull models, with time since landmark age as the time scale. Landmark age datasets were constructed comprising of participants with no CVD diagnoses and/or statin prescription prior to that landmark age and included the following variables: (i) landmark-age-dependent outcome time-to-CVD-event and censoring indicator; (ii) sex; (iii) landmark- age-dependent estimated error-free risk factor values for SBP, total cholesterol, HDL cholesterol, smoking status and the most recent observed records for diabetes status and history of blood pressure-lowering medication prescriptions, denoted together as $X(La)$ and (iv) landmark-age- dependent time-to-statin-initiation during follow-up (set to the "time-to-CVD-event" if not observed) and statin-initiation indicator. We then split the time-to-CVD-event records at the time-to-statin-initiation, so that individuals who had an indication of statin initiation during follow-up had two records, one covering the landmark age before statin initiation, and the second from statin initiation to the CVD event or censoring. To each landmark age data set, we fit the following sex-stratified Weibull model:

$$\mathrm{h}_S(t|X(La),La)= \mathrm{h}_{0S}(t)\exp[{\beta_x}^T X(La)+B\times Statin(t)]$$

where $\mathrm{h}_{0S}(t) = \lambda v t^{\nu-1}$, with scale and shape parameters λ and ν, and the scale parameter λ is parameterized as exp ($\beta 0$); $Statin(t)$ is the time dependent indicator which equals 0 before an indication of statin-initiation, and equals 1 at and after the first indication of statin-initiation; and B represents the effect of statin-initiation on the risk of CVD, which is constrained to B = ln (0.75) to represent a 25% risk reduction as reported from published meta-analyses of trials.[13,14] This is done by using *offset* option in the Weibull survival model in Stata. The code sample for each landmark age by gender for estimating 10-year CVD risk accounting for statin-initiation is as follows:

```
stset ft, failure (cvd_ind ==1) id(patid)
stsplit new_statin_ind, after(time=statin_time) at(0)
replace new_statin_ind=new_statin_ind+1
*convert -1, 0 to 0,1 for on statins
replace new_statin_ind=0 if statins_prscd==. |
```

```
(statins_prscd!=. & statins_prscd>=exit_date)
*for never statin before exit_date
gen beta_x=new_statin_ind*ln(0.75)
streg sbp bp_medication tchol hdl smoke diabetes if
derivation==1, offset(beta_x) dist(weibull)
```

where ft is follow-up time; cvd_ind is the incident CVD indicator; statin_time is the time-to-statin-initiation during follow-up (set to the "time-to-CVD-event" if not observed); new_statin_ind is the time-varying indicator for statin initiation (1 for on statins, 0 for no statins); ln(0.75) is the 25% risk reduction as reported from published meta-analyses of trials; *sbp, bp_medication, tchol, hdl, smoke, diabetes* are the risk factor values estimated from Stage 1.

Predicted 10-year CVD risk is estimated for participants at each landmark age from the equation:

$$1 - P(T > La + 10|T > La, X(La)) = 1 - S_{0S}(La + 10|La)^{\exp[\beta_x{}^T X(La) + B\times Statin(t)]}$$

where $S_{0S}(La + 10|La) = \exp(-\lambda t^{\nu})$ represents the sex-stratified 10-year baseline

survival from landmark age La.

Other survival models, including the non-parametric Cox model, and more flexible parametric forms, could be used in place of the Weibull model. We selected the Weibull model due to a reasonable fit, and to enable a closed form solution to the calculation of counterfactual survival times in the absence of statin initiation (see Appendix 3). In our analysis, the fitted survival probability curves from the Weibull models were consistent with the Kaplan—Meier curves, indicating a reasonable fit (Web Figure 4). The fitted Weibull model shape parameters ranged between 1.07 to 1.34 across landmark ages (Web Figures 5 and 6) the Weibull model was more sufficient than exponential model of which the shape parameter is defined as 1.

Proportional hazard assumption in Weibull model:

The Weibull hazard function is $h_0(t) = \lambda\nu t^{\nu-1}$ where the scale parameter λ is parametrized as $\exp(\beta_0)$. The hazard ratio for statin treatment is obtained as $HR =$

$\frac{\exp(\beta_0+\beta_S)\nu t^{\nu-1}}{\exp(\beta_0)\nu t^{\nu-1}} = \exp(\beta_S)$, which is the statin treatment effect. This result depends

on the shape parameter ν having the same value for treatment vs. non-treatment to be cancelled out to get HR, and so that the proportional hazard assumption is satisfied. In our analysis, the proportional hazard assumption was satisfied since the shape parameters are generally same for models ignoring vs. accounting for statin effect.

Text 2. Recalibration and rescaling of eHEART risks for population health modelling.

Our aim was to externally validate the *eHEART* tool to estimate the health impact in a general, English population. We used UK Biobank chosen due to its availability of detailed measurement at baseline, which was used to estimate a 10-year CVD risk using QRISK2 and represents a formal risk assessment, but also linked historical primary care electronic medical records necessary for prioritisation using *eHEART*.

However, UK Biobank participants has been shown to be healthier than the general population both in terms of risk factor levels and CVD incidence rates. Using *eHEART* and QRISK2 in UK Biobank without adjustments would lead to a biased distribution of 10-year risk estimated, with the distribution of risks being skewed to the right and be narrow relative to the distribution observed in the general population. To more accurately use UK Biobank for population health modelling, the distribution of 10-year risks estimated were adjusted using recalibration and rescaling.

Stage 1: recalibration

We first recalibrated both *eHEART* and QRISK2 to the UK Biobank population to ensure both tools were well calibrated to the population. This was done due to the different populations used to derive both risk tools and because of the different data types used in UK Biobank, with *eHEART* using the historical primary care records and QRISK2 using the baseline values. The methods used have been previously described.[15]

Recalibration was completed within each tool and sex, allowing the mean level of predicted risks to match what was observed. We used average risk factor levels calculated by 5-year age groups to estimate the predicted risk. For *eHEART*, the mean risk factor levels for continuous risk factors were calculated using the mean of the last observed values across all individuals with a primary care record before baseline, and for binary variables, the mean number of individuals with at least one positive record was used. For QRISK2, the mean value or prevalence of each risk factor at baseline was used. The observed risk was calculated using the CVD incidence rate of UK Biobank. A linear model was fit within each tool and sex to relate the observed risk (θ_{obs}) and predicted risk (θ_{pred}) estimated for each 5-year age group. (c_s):

$$\log_e(-\log_e(1-\theta_{obs,c_s})) = \beta_0 + \beta_1 \times \log_e(-\log_e(1-\theta_{pred,c_s}))$$

The estimated β_0 and β_1 were then used as scaling factors to recalibrate each individual's original 10-year risk ($\theta_{pred,i}$) to give a new recalibrated estimate $\theta_{newpred,i}$:

$$\theta_{newpred,i} = 1 - \exp(-\exp(\beta_0 + \beta_1 \times \log_e(-\log_e(1-\theta_{pred,i}))))$$

Stage 2: rescaling

Recalibrating both models using average risk factor values and incidence rates from UK Biobank resulted in the recalibrated 10-year risks to be heavily right skewed. To correct for this, we rescaled the recalibrated estimates to spread out the estimated risks to become more representative of what should be expected in the general population.

Rescaling was completed within each tool and sex. We calculated the mean recalibrated 10-year risk by age-group of UK Biobank to estimate the predicted risk. We then used mean risk factor levels calculated from the Clinical Practice Research Datalink (CPRD) between the years 2014 and 2019 within 5-year age groups to estimate the observed risk of using either tool in the general population. We then fit a linear model between the observed risk and the predicted risk using the same methods described in stage 1. The new scaling factors calculated were used to rescale each individual's recalibrated 10-year risk to give a new rescaled and recalibrated estimate.

Stage 3: Population health modelling

We created a hypothetical population of 100,000 individuals (50,000 men and women. We used data from the ONS to approximate the age structure of the general English population. We estimated the expected number of events observed in the hypothetical population using CVD incidence rates estimated from individuals with at least one primary care record of systolic blood pressure, total or HDL cholesterol or smoking status in CPRD between the years 2014 and 2019.

To estimate the number needed to screen (NNS) to prevent one CVD event, we first calculated the proportion of cases in UK Biobank that were deemed high risk (10-year risk >= 10%) by QRISK2 within 5-year age groups and sex. We used this proportion to estimate the number of CVD events identified in the hypothetical population, and applied a HR of 0.8 to model the benefits of statin initiation, with the number of events saved was defined as the difference in the number of events after statin initiation and the initial expected number of events. The NNS was calculated by taking the number of individuals assessed and dividing by the number of events saved. The NNS after using *eHEART* to prioritise individuals for a formal assessment was calculated in a similar manner, where the number of individuals formally assessed included only those prioritised using *eHEART*. 95% confidence intervals for the NNS were estimated using the empirical bootstrap method with 1000 iterations for each age-group and sex. Age and sex specific prioritisation thresholds were chosen to optimise the false negative rate of QRISK2 in UK Biobank.

Text 3. Derivation of QRISK2 prioritisation tool for population health modelling

Prioritisation of 10-year CVD risks using an estimated value of QRISK2 were calculated using last observed values within primary care records at the baseline age of UK Biobank and published coefficients. Ethnicity and deprivation information was obtained using the self-reported baseline data. Missing values for SBP, cholesterol levels and BMI were imputed using age-, sex- and ethnicity-specific means estimated in UK Biobank[16]. The range of valid measurements for QRISK2 mirrors the range used in the published calculator and were restricted to: SBP 70 to 210 mmHg; cholesterol ratio 1 to 12; BMI 20 to 40kg /m^2.

References

1. Hippisley-Cox J, Coupland C, Brindle P. Development and validation of QRISK3 risk prediction algorithms to estimate future risk of cardiovascular disease: Prospective cohort study. BMJ [Internet]. 2017 May 23 [cited 2021 Jan 18];357. Available from: http://www.bmj.com/

2. Herrett E, Shah AD, Boggon R, Denaxas S, Smeeth L, Van Staa T, et al. Completeness and diagnostic validity of recording acute myocardial infarction events in primary care, hospital care, disease registry, and national mortality records: cohort study. BMJ [Internet]. 2013 May 21 [cited 2022 Feb 25];346(7909). Available from: https://www.bmj.com/content/346/bmj.f2350

3. Paige E, Barrett J, Stevens D, Keogh RH, Sweeting MJ, Nazareth I, et al. Landmark Models for Optimizing the Use of Repeated Measurements of Risk Factors in Electronic Health Records to Predict Future Disease Risk. Am J Epidemiol [Internet]. 2018 Jul 1 [cited 2021 Nov 23];187(7):1530–8. Available from: https://academic.oup.com/aje/article/187/7/1530/4952104

4. Goff DC, Lloyd-Jones DM, Bennett G, Coady S, D'Agostino RB, Gibbons R, et al. 2013 ACC/AHA guideline on the assessment of cardiovascular risk: A report of the American college of cardiology/American heart association task force on practice guidelines. Circulation [Internet]. 2014 Jun 24 [cited 2022 Feb 25];129(25 SUPPL. 1):49–73. Available from: https://www.ahajournals.org/doi/abs/10.1161/01.cir.0000437741.48606.98

5. Sharma M, Petersen I, Nazareth I, Coton SJ. An algorithm for identification and classification of individuals with type 1 and type 2 diabetes mellitus in a large primary care database. Clin Epidemiol [Internet]. 2016 Oct 12 [cited 2021 Nov 23];8:373–80. Available from: https://pubmed.ncbi.nlm.nih.gov/27785102/

6. Verbeke G, Fieuws S, Molenberghs G, Davidian M. The analysis of multivariate longitudinal data: a review. Stat Methods Med Res [Internet]. 2014 Feb [cited 2022 Feb 25];23(1):42–9. Available from: https://pubmed.ncbi.nlm.nih.gov/22523185/

7. Schafer JL. Analysis of Incomplete Multivariate Data, 1st edition , New York: Chapman and Hall/CRC. Chapman and Hall/CRC, editor. 1997;444.

8. Wood AM, Thompson SG, Kostis JB, Wilson AC, Wu K, Benderly M, et al. Correcting for multivariate measurement error by regression calibration in meta-analyses of epidemiological studies. Stat Med [Internet]. 2009 Mar 30 [cited 2022 Feb 25];28(7):1067–92. Available from: https://pubmed.ncbi.nlm.nih.gov/19222086/

9. White I, Frost C, Tokunaga S. Correcting for measurement error in binary and continuous variables using replicates. Stat Med [Internet]. 2001 Nov 30 [cited 2022 Feb 25];20(22):3441–57. Available from: https://pubmed.ncbi.nlm.nih.gov/11746328/

10. Goldberger AS. Best Linear Unbiased Prediction in the Generalized Linear Regression Model. J Am Stat Assoc. 1962 Jun;57(298):369.

11. Goldstein H. Multilevel Statistical Models. 2010 Oct 29 [cited 2022 Feb 25]; Available

from: https://onlinelibrary.wiley.com/doi/book/10.1002/9780470973394

12. Diggle P, Diggle P. Analysis of longitudinal data. 2002;379.

13. Cook NR, Ridker P. Further insight into the cardiovascular risk calculator: the roles of statins, revascularizations, and underascertainment in the Women's Health Study. JAMA Intern Med [Internet]. 2014 Dec 1 [cited 2022 Feb 17];174(12):1964–71. Available from: https://pubmed.ncbi.nlm.nih.gov/25285455/

14. Mihaylova B, Emberson J, Blackwell L, Keech A, Simes J, Barnes EH, et al. The effects of lowering LDL cholesterol with statin therapy in people at low risk of vascular disease: Meta-analysis of individual data from 27 randomised trials. Lancet [Internet]. 2012 Aug 1 [cited 2021 Nov 23];380(9841):581–90. Available from: http://www.thelancet.com/article/S0140673612603675/fulltext

15. Pennells L, Kaptoge S, Wood A, Sweeting M, Zhao X, White I, et al. Equalization of four cardiovascular risk algorithms after systematic recalibration: Individual-participant meta-analysis of 86 prospective studies. Eur Heart J. 2019;40(7):621–31.

16. Pate A, Emsley R, Ashcroft DM, Brown B, Van Staa T. The uncertainty with using risk prediction models for individual decision making: An exemplar cohort study examining the prediction of cardiovascular disease in English primary care. BMC Med [Internet]. 2019 Jul 17 [cited 2020 Apr 27];17(1):134. Available from: https://bmcmedicine.biomedcentral.com/articles/10.1186/s12916-019-1368-8

17. Benchimol EI, Smeeth L, Guttmann A, Harron K, Moher D, Peteresen I, et al. The REporting of studies Conducted using Observational Routinely-collected health Data (RECORD) Statement. PLOS Med [Internet]. 2015 [cited 2021 Nov 23];12(10):e1001885. Available from: https://journals.plos.org/plosmedicine/article?id=10.1371/journal.pmed.1001885

18. Collins GS, Reitsma JB, Altman DG, Moons KGM. Transparent Reporting of a Multivariable Prediction Model for Individual Prognosis or Diagnosis (TRIPOD): The TRIPOD Statement. Circulation [Internet]. 2015 [cited 2021 Nov 24];131(2):211. Available from: /pmc/articles/PMC4297220/
